# Endophytes of *Withania somnifera* modulate *in planta* content and the site of withanolide biosynthesis

**DOI:** 10.1038/s41598-018-23716-5

**Published:** 2018-04-03

**Authors:** Shiv S. Pandey, Sucheta Singh, Harshita Pandey, Madhumita Srivastava, Tania Ray, Sumit Soni, Alok Pandey, Karuna Shanker, C. S. Vivek Babu, Suchitra Banerjee, M. M. Gupta, Alok Kalra

**Affiliations:** 1 Microbial Technology Department, CSIR-Central Institute of Medicinal and Aromatic Plants, Lucknow, 226015 India; 2 Plant Biotechnology Division, CSIR-Central Institute of Medicinal and Aromatic Plants, Lucknow, 226015 India; 3 Analytical Chemistry Department, CSIR-Central Institute of Medicinal and Aromatic Plants, Lucknow, 226015 India; 4 CSIR-Central Institute of Medicinal and Aromatic Plants, Research Centre, Allalasandra, GKVK Post, Bangalore, 560065 India

## Abstract

Tissue specific biosynthesis of secondary metabolites is a distinguished feature of medicinal plants. *Withania somnifera*, source of pharmaceutically important withanolides biosynthesizes withaferin-A in leaves and withanolide-A in roots. To increase the *in planta* withanolides production, a sustainable approach needs to be explored. Here, we isolated endophytes from different parts of *W. somnifera* plants and their promising role in *in planta* withanolide biosynthesis was established in both *in-vivo* grown as well in *in-vitro* raised composite *W. somnifera* plants. Overall, the fungal endophytes improved photosynthesis, plant growth and biomass, and the root-associated bacterial endophytes enhanced the withanolide content in both *in-vivo* and *in-vitro* grown plants by modulating the expression of withanolide biosynthesis genes in leaves and roots. Surprisingly, a few indole-3-acetic acid (IAA)-producing and nitrogen-fixing root-associated endophytes could induce the biosynthesis of withaferin-A in roots by inducing *in planta* IAA-production and upregulating the expression of withanolide biosynthesis genes especially MEP-pathway genes (*DXS* and *DXR*) in roots as well. Results indicate the role of endophytes in modulating the synthesis and site of withanolides production and the selected endophytes can be used for enhancing the *in planta* withanolide production and enriching roots with pharmaceutically important withaferin-A which is generally absent in roots.

## Introduction

*Withania somnifera* well-known as Indian ginseng or Ashwagandha, is an important medicinal plant widely distributed around the globe and used in traditional medicine systems like Ayurveda, Siddha, Unani and Chinese. All parts of this plant like roots, stem, bark, leaves, flowers and seeds have medicinal importance^1,2^. Medicinal properties of *W. somnifera* include anti-hyperglycemic, neuropharmacological, immunomodulatory, cardioprotective, musculotropic, hepatoprotective, radiosensitizing, chemoprotective, anti-aging, macrophage-activating, diuretic, hypocholesterolemic, aphrodisiac, rejuvenating and hemopoietic^3–7^. *Withania* leaves are used in the treatment of fever, tumours and ulcers^8^. Medicinal importance of different parts of *W. somnifera* is due to the presence of pharmaceutically active steroidal lactones withanolides including withanolide-A and withaferin-A as major bioactive molecules. Withaferin-A has anti-leukemic, anti-invasive, anti-metastatic, apoptotic, anti-inflammatory, radiosensitizing and antidiabetic activity and also act as a potential leptin-sensitizer^9–12^. Withanolide-A is a potential neurological, immunological and anti-stress agent^13–15^. Roots of *W. somnifera* plants are rich in withanolide-A however withaferin-A is present in leaves in large amount and totally absent or present in traces in roots of *W. somnifera* plant^16–20^. Withanolides are terpenoids and synthesised in plants using the precursor isoprenoids which are synthesized via mevalonate (MVA) and 2-C-methyl-D-erythritol-4-phosphate (MEP) pathway. Genes encoding key enzymes of withanolide biosynthesis have been characterized^21,22^ and consistent efforts are being made to increase the production of withanolides in roots as well as leaves. Biotechnological tools like genetic engineering are being focused for enhancing the withanolide production. Overexpression of squalene synthase (*SQS*), a key regulatory gene of withanolide biosynthesis in *W. somnifera* could increase the content of withaferin A and withanolide A in the leaves up to 4–4.5 fold^23^. Overexpression of cycloartenol synthase (*CAS*) in *W. somnifera* increased the withanolide content to the extent of 1.06 to 1.66 fold^24^. Use of cell suspension^25^, adventitious^26,27^, and hairy root culture^28^ for improvement of withanolide production have also been tried. Overexpression of *SQS* in the *Withania* suspension cultures enhanced the withanolide A content up to 2.5 fold compared to non-transformed culture^29^. Enhanced production of withanolides could be achieved by the treatment of salicylic acid and methyl jasmonate in *Withania* hairy roots^28^. It has been observed that treatment with the extract of sea weeds on *Withania* hairy roots increased the production of withanolides by increasing the expression of key genes of withanolide biosynthesis such as squalene epoxidase (*SQE*), *SQS*, 3-hydroxy-3-methylglutaryl-coenzyme A reductase (*HMGR*) and farnesyl pyrophosphate synthase (*FPPS*)^30^. Generation of transgenics by genetic manipulation of withanolide biosynthetic pathway and the cell culture approaches have limitations related to practical and economic feasibility and social acceptability, therefore, requires exploration of new sustainable approach for enhancement of withanolide production.

Endophytes are known to promote plant growth, protect plants to environmental stresses, and as the source of therapeutic compounds^31–38^. Endophytes are found to be associated with different parts of a plant and are involved in modulation of primary and secondary metabolism of the host plant^39,40^. Occasionally, endophytes are also found to be involved in improving secondary metabolite biosynthesis of the host plant^39–43^. Therefore, endophyte-mediated improvement of crop yield and secondary metabolite biosynthesis of medicinal plants could be a sustainable approach for producing the higher yield of therapeutically important chemicals. In the present study, efforts were made to identify the most potent endophytes from *W. somnifera* plants to decipher their roles in enhancing the plant growth and in parallel, the overall yield of therapeutically important withanolides, through their judicious screening on *in-vivo* grown plants as well as in *in-vitro* raised composite plants (i.e., plants with wild-type shoots and wild type *Agrobacterium rhizogenes* induced transgenic roots, as illustrated previously^44^). Since *A. rhizogenes* is reputed to stimulate robust root formation upon infection and roots of *W. somnifera* plants are considered as the major sites of its desired metabolite synthesis, the envisaged rationale of its use in raising the composite plants during the present course of study imparts the overall intention of expanding the host-endophyte association prospect for better comprehension of the plant-endophyte interaction. To the best of our knowledge, the use of composite plant for serving this specific goal remained uncharted so far. Instead, composite plants had earlier been successfully utilized as a suitable alternative for the functional characterization of any targeted gene(s) in the host plants to overcome their prevailing tedious and lengthy transformation and regeneration processes^45^. Then again, several other studies, such as nutrient/hormone uptake, interactions with root nodulating bacteria and mycorrhizal symbiotic association, have also documented the competence of this composite plant based technology^46^.

## Results

### Isolation and molecular characterization of endophytes

Endophytes were isolated from leaves, roots and seeds of *W. somnifera* plant. We performed 16S rRNA and ITS sequencing for identification of bacterial and fungal endophytes, respectively. A total of 40 isolates including 29 bacteria and 11 fungi were isolated and identified (Table 1); of these 25 endophytes (18 bacteria and 7 fungi) from roots, 12 endophytes (8 bacteria and 4 fungi) from leaves and 3 bacterial endophytes from seeds were isolated.

**Table 1:** List of endophytes isolated from *Withania somnifera* cv. Poshita.

Plant part	Strain	Culture name	Accession no.
**Bacterial endophytes**			
Leaves	WPL1	*Bacillus amyloliquefaciens*	KY000380
	WPL2	*Bacillus horneckiae*	KY000381
	WPL3	*Staphylococcus haemolyticus*	KY000382
	WPL4	*Bacillus* sp.	KY000383
	WPL5	*Micrococcus luteus*	KY000384
	WPL6	*Pseudomonas putida*	KY000385
	WPL7	*Bacillus firmus*	KY000386
	WPL8	*Bacillus licheniformis*	KY000387
Root	WPR12	*Bacillus muralis*	KY000391
	WPR13	*Brevibacterium frigoritolerans*	KY000392
	WPR14	*Bacillus pumilis*	KY000393
	WPR15	*Bacillus aryabhattai*	KY000394
	WPR16	*Bacillus megaterium*	KY000395
	WPR17	*Pseudomonas* sp.	KY000396
	WPR18	*Bacillus pseudomycoides*	KY000397
	WPR19	*Bacillus aquimaris*	KY000398
	WPR20	*Bacillus thuringiensis*	KY000399
	WPR21	*Bacillus indicus*	KY000400
	WPS23	*Streptomyces* sp.	KY454621
	WPR26	*Paenibacillus* sp.	KY000401
	WPR27	*Bacillus cereus*	KY000402
	WPR28	*Bacillus thuringiensis*	KY000403
	WPR29	*Pseudomonas* sp.	KY000404
	WPR30	*Rhizobium sullae*	KY000405
	WPR31	*Sinorhizobium fredii*	KY000406
	WPR32	*Pantoea* sp.	KY000407
Seed	WPS9	*Bacillus subtilis*	KY000388
	WPS10	*Bacillus cereus*	KY000389
	WPS11	*Bacillus tequilensis*	KY000390
**Fungal endophytes**			
Leaves	WPLF1	*Penicillium* sp.	KX928761
	WPLF2	*Aspergillus terreus*	KX928762
	WPLF3	*Trametes versicolor*	KX928763
	WPLF4	*Sarocladium implicatum*	KX928764
Root	WPRF5	*Penicillium oxalicum*	KX928765
	WPRF6	*Colletotrichum capsici*	KX928766
	WPRF7	*Ceratobasidium* sp.	KX928767
	WPRF8	*Penicillium* sp.	KX928768
	WPRF9	*Aspergillus brasiliensis*	KX928769
	WPRF10	*Colletotrichum truncatum*	KX928770
	WPRF11	*Hypocrea lixii*	KX928771

### Photosynthetic pigments, photosynthesis, stomatal conductance and transpiration rate

To study the individual effect of a particular endophyte on photosynthetic efficiency of *W. somnifera* plants, each endophyte was inoculated to the endophyte-free plants and their photosynthetic pigment content, net CO_2_ assimilation, transpiration rate and stomatal conductance were measured, and compared with the non-inoculated endophytes free control plants. Interestingly, inoculation with fungal endophytes, in general, improved the photosynthetic efficiency of *W. somnifera* plants. WPLF4, WPRF5, WPRF6, WPRF7, WPRF8, WPRF9, WPRF10, WPRF11 inoculated plants had higher chlorophyll (17–29%), carotenoid (29–46%), net CO_2_ assimilation (17–43%), transpiration rate (18–31%) and stomatal conductance (10–42%) than that of non-inoculated endophyte-free control plants (Table 2). WPLF2 and WPLF3 inoculated plants had 16.4% and 22.4% higher transpiration rate respectively than that of non-inoculated endophyte free control plants. WPLF3 inoculation could also increase net CO_2_ assimilation by 12.2% compared to endophyte free control plants. Inoculation with bacterial endophytes could not improve the measured photosynthesis parameters significantly (Supplementary Table S1).

**Table 2:** Effect of inoculation with fungal endophytes on physiological parameters of *Withania somnifera* plants.

Treatment	Chlorophyll (mg gFW^−1^)	Carotenoids (mg gFW^−1^)	A (μ mol m^−2^ s^−1^)	E (m mol m^−2^ s^−1^)	gS (m mol m^−2^ s^−1^)
Control	0.550 ± 0.009^d^	0.087 ± 0.006^b^	23.50 ± 0.35^e^	10.98 ± 0.51^c^	498.00 ± 9.81 ^f^
WPLF1	**0.608 ±** **0.039**^**bc**^	0.094 ± 0.004^b^	25.50 ± 1.09^de^	11.22 ± 0.60^c^	510.00 ± 21.55^ef^
WPLF2	0.595 ± 0.017 ^cd^	0.084 ± 0.005^b^	25.27 ± 0.78^de^	**12.77 ± 0.51** ^**b**^	514.00 ± 9.29^ef^
WPLF3	**0.603 ± 0.010** ^**bc**^	0.091 ± 0.005^b^	**26.37 ± 0.33** ^**d**^	**13.43 ± 0.44** ^**ab**^	489.67 ± 3.84 ^f^
WPLF4	**0.646 ± 0.011** ^**ab**^	**0.111 ± 0.007** ^**a**^	**27.93 ± 0.88** ^**bc**^	**13.04 ± 0.45** ^**ab**^	**629.00 ± 13.45** ^**bc**^
WPRF5	**0.663 ± 0.019** ^**ab**^	**0.117 ± 0.007** ^**a**^	**27.53 ± 0.44 ** ^**cd**^	**14.02 ± 0.42** ^**ab**^	**645.00 ± 9.87** ^**b**^
WPRF6	**0.701 ± 0.048** ^**a**^	**0.116 ± 0.008** ^**a**^	**30.83 ± 0.87** ^**ab**^	**13.20 ± 0.17** ^**ab**^	**663.67 ± 17.91** ^**ab**^
WPRF7	**0.712 ± 0.027** ^**a**^	**0.122 ± 0.004** ^**a**^	**30.20 ± 1.49** ^**bc**^	**13.31 ± 0.32** ^**ab**^	**592.00** ± **24.50 **^**cd**^
WPRF8	**0.692 ± 0.023** ^**a**^	**0.123 ± 0.002** ^**a**^	**33.60 ± 0.55** ^**a**^	**14.35 ± 0.60** ^**a**^	**707.00 ± 13.53** ^**a**^
WPRF9	**0.695 ± 0.011** ^**a**^	**0.127 ± 0.002** ^**a**^	**31.03 ± 1.86** ^**ab**^	**13.02 ± 0.33** ^**ab**^	**664.67** **±** **9.70**^**ab**^
WPRF10	**0.678 ± 0.022** ^**ab**^	**0.126 ± 0.005** ^**a**^	**30.30 ± 1.16** ^**bc**^	**12.97 ± 0.52** ^**ab**^	**574.33 ± 2.85** ^**d**^
WPRF11	**0.703 ± 0.013** ^**a**^	**0.125 ± 0.005** ^**a**^	**28.10 ± 0.90** ^**bc**^	**13.23 ± 0.43** ^**ab**^	**548.00 ± 27.02** ^**d**e^

A-Net CO_2_ assimilation, E-Transpiration rate, gS-Stomatal conductance. Values are the means of six biological replicates ± S.E. Values with different letters are significantly different at *P* ≤ 0.05 (Duncan’s multiple range test). Values significantly higher than that of control plants are highlighted in bold.

### Plant biomass

**Figure 1:**
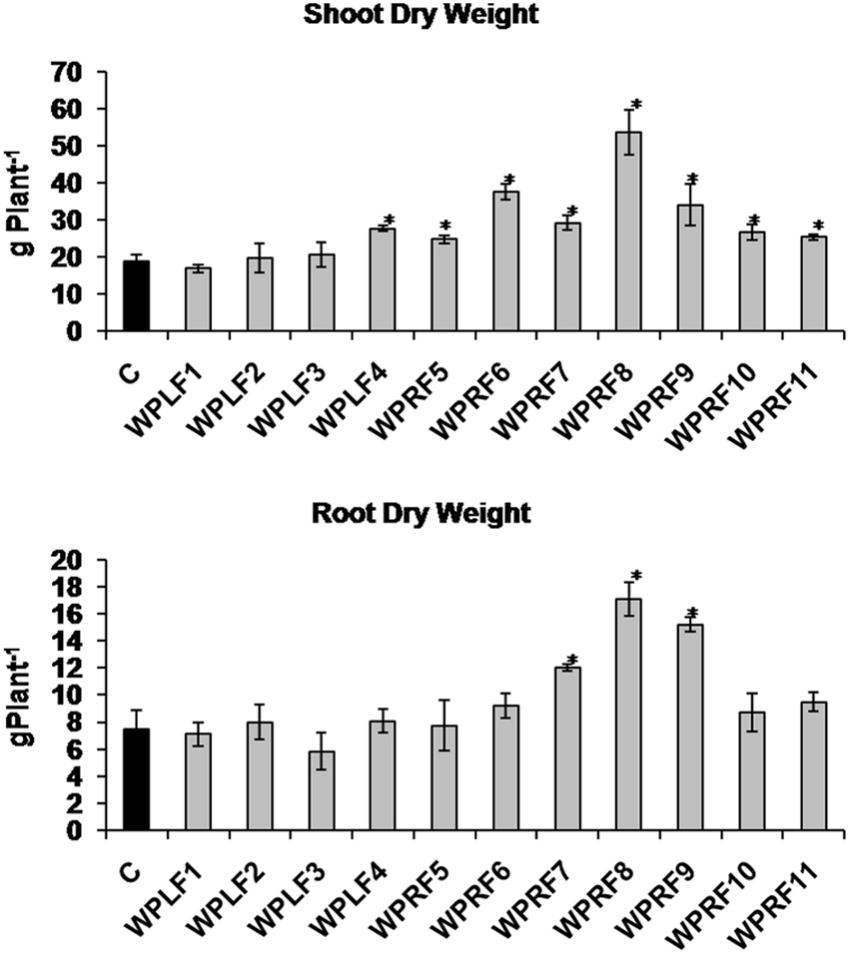
Effect of endophyte-inoculation on shoot and root biomass of *Withania somnifera* plant. One month old endophyte-free *W. somnifera* plants that originated from seeds treated with bactericides and fungicides were inoculated with endophytes and grown in pots filled with autoclaved soil and vermicompost mixture. Non-inoculated endophyte-free plants were used as a control. At 90 days stage shoot and root biomass was measured. Each data point is an average of six replicates and the error bars represent standard errors. Asterisks indicate significant differences between the control and endophyte inoculation (Duncan’s multiple range test *P* ≤ 0.05).

Some of the isolated fungal endophytes were able to enhance the shoot and root biomass of *W. somnifera* (Fig. 1). It was observed that inoculation with isolated bacterial endophytes could not improve the shoot and root biomass of *W. somnifera* plants significantly (Supplementary Table S2). WPLF4, WPRF5, WPRF6, WPRF7, WPRF8, WPRF9, WPRF10 and WPRF11 inoculated plants had 32–189% higher shoot biomass than that of non-inoculated endophyte free control plants (Fig. 1). Inoculation with WPRF7, WPRF8, and WPRF9 could also enhance the root biomass by 62–130% (Fig. 1). The endophytes, WPRF7, WPRF8 and WPRF9 were found to be effective in enhancing both shoot and root biomass.

### Plant growth promotion activity of selected endophytes

Plant growth promotion traits such as phosphate solubilization and indole acetic acid (IAA) production was tested in the selected bacterial and fungal endophytes able to promote the growth of the *W. somnifera* plant and enhance the content of withanolides. Fungal endophytes WPLF1, WPLF2, WPLF3, WPRF5, WPRF7, WPRF8, WPRF9 and WPRF10 were found positive for phosphate solubilization and WPLF3, WPRF6, WPRF7, WPRF9, WPRF10 and WPRF11were positive for IAA production (Supplementary Table S3). Among bacterial endophytes, WPR12, WPR16, WPR17, WPS23 and WPR32 were found positive for IAA production and nitrate reduction activity, and negative for phosphate solubilization (Supplementary Table S4).

### Withanolides

Effect of endophyte inoculation on withanolide production was studied by measuring the content of withaferin A (WFA), 12-deoxy withstramonolide (DWL) and withanolide A (WLA) in leaves and roots of *Withania* plant. Endophytes (WPS11, WPR13, WPR16, WPR17, WPR19, WPR20, WPS23, WPR26, WPR27, WPR28, WPR30, WPR31, WPR32, WPLF1, WPLF2, WPLF4, WPRF5, WPRF6, WPRF7, WPRF9 and WPRF11) inoculated *Withania* plants had 20–232% higher WFA content in leaves compared to non-inoculated endophyte free control plants (Table 3). WPL3, WPS10, WPR12, WPR21 inoculated plants had 108–383% higher leaf DWL content. Endophyte inoculations, however, could not improve the WLA content in leaves of *Withania* plant significantly, interestingly, however, some endophyte-inoculations (WPL5, WPL6, WPL7, WPR12, WPR16, WPR17, WPR18, WPS23, WPR28, WPR32 and WPLF1) induced the biosynthesis of WFA in roots which could not be detected in non-inoculated endophyte free control plants (Table 3). On the other hand, DWL which could not be detected in roots of endophyte-free control *Withania* plants was detected in roots of WPL2, WPL3, WPL4, WPL5, WPS10, WPR12, WPR17 inoculated plants (Table 3). In roots, WLA content enhanced considerably by 75–298% with the inoculation of few endophyte (WPL1, WPL4, WPS10, WPR15, WPR18, WPR21, WPS23, WPR26, WPR29, WPR30, WPR31, WPR32, WPLF1, WPLF2 and WPRF10). None of the endophytes produced secondary metabolites of the host plant in culture medium i.e. independent of the host (data not shown).

**Table 3:** Withanolide content in leaves and roots of endophyte-inoculated *Withania* plants.

Treatment	Leaf			Root		
	%WFA	%DWL	%WLA	%WFA	%DWL	%WLA
Control	0.56 ± 0.04^hi^	0.12 ± 0.009^ef^	0.35 ± 0.029^ab^	nd	nd	0.067 ± 0.012^gh^
WPL1	0.39 ± 0.05 ^lm^	0.09 ± 0.009^ef^	0.21 ± 0.035^hi^	nd	nd	**0.143 ± 0.015 ** ^**cd**^
WPL2	0.32 ± 0.09^mn^	0.03 ± 0.009^ij^	0.04 ± 0.006^p^	nd	**0.012 ± 0.002** ^**d**^	0.043 ± 0.009^hi^
WPL3	0.50 ± 0.07^ij^	**0.43 ± 0.055** ^**b**^	0.09 ± 0.006^op^	nd	**0.013 ± 0.003** ^**d**^	0.070 ± 0.010^gh^
WPL4	0.52 ± 0.12^ij^	0.09 ± 0.009^ef^	0.35 ± 0.026^ab^	nd	**0.013 ± 0.003** ^**d**^	**0.177 ± 0.009** ^**bc**^
WPL5	0.58 ± 0.14^gh^	0.13 ± 0.019^e^	0.27 ± 0.067 ^cd^	**0.013 ± 0.002** ^**d**^	**0.012 ± 0.002** ^**d**^	0.067 ± 0.012^gh^
WPL6	0.56 ± 0.13^hi^	0.01 ± 0.003^j^	0.08 ± 0.007^op^	**0.012 ± 0.002** ^**d**^	nd	0.063 ± 0.009^hi^
WPL7	0.53 ± 0.05^ij^	0.13 ± 0.009^ef^	0.25 ± 0.043^de^	**0.015 ± 0.003 ** ^**cd**^	nd	0.073 ± 0.007^gh^
WPL8	0.58 ± 0.15^gh^	0.07 ± 0.010^ef^	0.40 ± 0.086^a^	nd	nd	0.057 ± 0.012^hi^
WPS9	0.48 ± 0.15^ij^	0.13 ± 0.015^e^	0.20 ± 0.041^hi^	nd	nd	0.053 ± 0.012^hi^
WPS10	0.59 ± 0.10^gh^	**0.58 ± 0.067** ^**a**^	0.44 ± 0.086^a^	nd	**0.022 ± 0.004** ^**b**^	**0.170 ± 0.015** ^**bc**^
WPS11	**0.85 ± 0.03 ** ^**cd**^	0.13 ± 0.018^e^	0.22 ± 0.018^gh^	nd	nd	0.060 ± 0.006^hi^
WPR12	0.15 ± 0.04^n^	**0.25 ± 0.018** ^**d**^	nd	**0.012 ± 0.002** ^**d**^	**0.018 ± 0.002** ^**c**^	0.077 ± 0.015^gh^
WPR13	**1.31 ± 0.17** ^**b**^	0.11 ± 0.015^ef^	Nd	nd	nd	0.060 ± 0.010^hi^
WPR14	0.58 ± 0.12^gh^	0.07 ± 0.015^ef^	0.17 ± 0.009^hi^	nd	nd	0.053 ± 0.009^hi^
WPR15	0.45 ± 0.09^ij^	0.05 ± 0.012^hi^	0.23 ± 0.012^fg^	nd	nd	**0.130 ± 0.015** ^**de**^
WPR16	**0.98 ± 0.10** ^**bc**^	0.08 ± 0.003^ef^	0.18 ± 0.019^hi^	**0.043 ± 0.003** ^**a**^	nd	0.073 ± 0.009^gh^
WPR17	**1.86 ± 0.15** ^**a**^	0.07 ± 0.015^ef^	0.42 ± 0.075^a^	**0.012 ± 0.002** ^**d**^	**0.038** ± **0.002**^**a**^	0.070 ± 0.012^gh^
WPR18	0.44 ± 0.17^jl^	0.11 ± 0.015^ef^	0.26 ± 0.045 ^cd^	**0.015 ± 0.003 ** ^**cd**^	nd	**0.183 ± 0.026** ^**bc**^
WPR19	**1.00 ± 0.08** ^**bc**^	0.09 ± 0.012^ef^	0.24 ± 0.007^ef^	nd	nd	0.060 ± 0.012^hi^
WPR20	**1.05 ± 0.06** ^**bc**^	0.06 ± 0.018^ef^	0.26 ± 0.009 ^cd^	nd	nd	0.067 ± 0.009^gh^
WPR21	0.56 ± 0.10^hi^	**0.32 ± 0.078** ^**c**^	0.16 ± 0.020^ij^	nd	nd	**0.117 ± 0.013** ^**ef**^
WPS23	**0.79 ± 0.06** ^**de**^	0.06 ± 0.010^fg^	0.14 ± 0.012 ^lm^	**0.018 ± 0.002** ^**bc**^	nd	**0.267 ± 0.018** ^**a**^
WPR26	**0.67 ± 0.14** ^**fg**^	0.08 ± 0.006^ef^	0.33 ± 0.026^ab^	nd	nd	**0.157 ± 0.024 ** ^**cd**^
WPR27	**0.75 ± 0.06** ^**ef**^	0.09 ± 0.006^ef^	0.11 ± 0.012^mn^	nd	nd	0.043 ± 0.012^hi^
WPR28	**1.11 ± 0.09** ^**bc**^	0.09 ± 0.012^ef^	0.23 ± 0.021^fg^	**0.022 ± 0.004** ^**b**^	nd	0.060 ± 0.015^hi^
WPR29	0.41 ± 0.13 ^lm^	0.08 ± 0.010^ef^	0.39 ± 0.032^ab^	nd	nd	**0.217 ± 0.027** ^**b**^
WPR30	**1.05 ±** **0.08**^**bc**^	0.10 ± 0.006^ef^	0.15 ± 0.015^jl^	nd	nd	**0.160 ±** **0.015 **^**cd**^
WPR31	**0.94 ±** **0.06**^**bc**^	0.09 ± 0.006^ef^	0.19 ± 0.010^hi^	nd	nd	**0.160 ± 0.015 ** ^**cd**^
WPR32	**1.17 ±** **0.13**^**bc**^	0.07 ± 0.009^ef^	0.28 ± 0.015^bc^	**0.010 ±** **0.001**^**d**^	nd	**0.117 ±** **0.022**^**ef**^
WPLF1	**0.73 ± 0.11** ^**ef**^	0.06 ± 0.015^gh^	0.27 ± 0.030 ^cd^	**0.012 ±** **0.002**^**d**^	nd	**0.130 ±** **0.015**^**de**^
WPLF2	**0.71 ±** **0.11**^**fg**^	0.06 ± 0.009^fg^	0.28 ± 0.021^bc^	nd	nd	**0.127 ±** **0.023**^**de**^
WPLF3	0.62 ± 0.13^gh^	0.08 ± 0.006^ef^	0.37 ± 0.029^ab^	nd	nd	0.073 ± 0.019^gh^
WPLF4	**1.20 ±** **0.10**^**bc**^	0.12 ± 0.012^ef^	0.22 ± 0.023^gh^	nd	nd	0.027 ± 0.009^ij^
WPRF5	**0.85 ±** **0.18 **^**cd**^	0.12 ± 0.017^ef^	0.18 ± 0.015^hi^	nd	nd	0.060 ± 0.006^hi^
WPRF6	**1.03 ±** **0.13**^**bc**^	0.10 ± 0.009^ef^	0.34 ± 0.035^ab^	nd	nd	0.057 ± 0.018^hi^
WPRF7	**0.68 ±** **0.10**^**fg**^	0.09 ± 0.003^ef^	0.27 ± 0.061 ^cd^	nd	nd	0.030 ± 0.012^ij^
WPRF8	0.43 ± 0.12 ^lm^	0.03 ± 0.009^ij^	0.37 ± 0.045^ab^	nd	nd	0.050 ± 0.006^hi^
WPRF9	**0.68 ±** **0.11**^**fg**^	0.09 ± 0.009^ef^	0.11 ± 0.026^no^	nd	nd	0.043 ± 0.018^hi^
WPRF10	0.46 ± 0.16^ij^	0.06 ± 0.017^fg^	0.22 ± 0.028^gh^	nd	nd	**0.177** ± **0.037**^**bc**^
WPRF11	**0.69 ±** **0.12**^**fg**^	0.11 ± 0.010^ef^	0.25 ± 0.022^ef^	nd	nd	0.047 ± 0.018^hi^

WFA- withaferin A, DWL- 12-deoxy withstramonolide and WLA- withanolide A. Values are the means of six biological replicates ± S.E. Values with different letters are significantly different at *P ≤ *0.05 (Duncan’s multiple range test).Values significantly higher than that of control plants are highlighted in bold. nd- not detected.

### Quantitative Real-time PCR (qRT-PCR) analysis of withanolide biosynthetic genes

**Figure 2:**
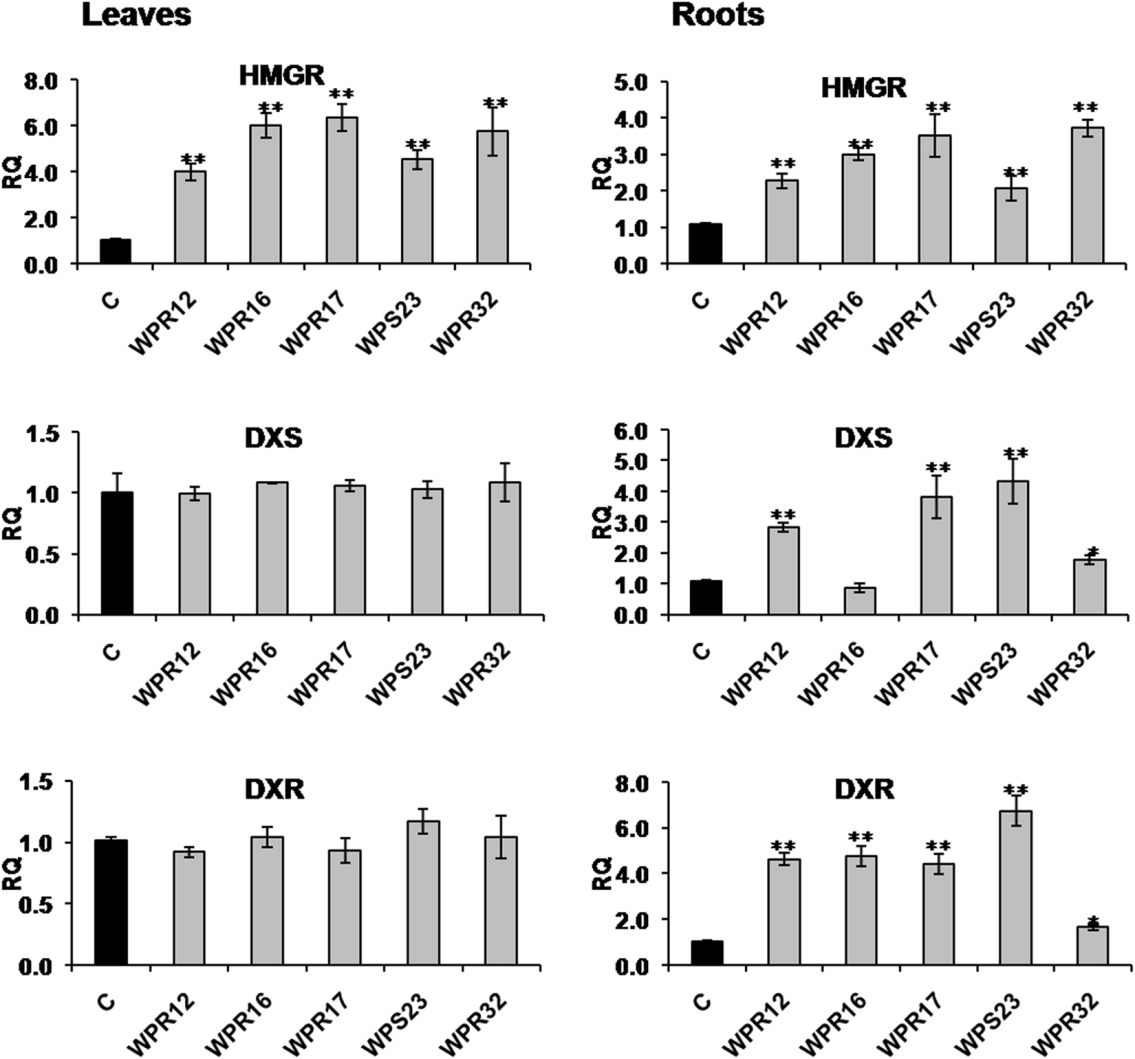
Effect of endophyte inoculation on the expression of genes involved in Mevalonate (MVA) and 2-C-methyl-D-erythritol-4-phosphate (MEP) pathway. Expression of MVA pathway gene (3-hydroxy-3-methylglutaryl-coenzyme A reductase; *HMGR*), and MEP pathway genes (1-Deoxy-D-xylulose-5-phosphate synthase; *DXS* and 1-deoxy-D-xylulose-5-phosphate reductase; *DXR*) was analyzed. Results were normalized to actin (reference transcript) and are shown relative to the level in non-inoculated endophyte-free control plants (calibrator). Data are means ± SD (*n* = 3 biological replicates) and *Y*-axis represents relative quantity (RQ). Asterisks indicate significant differences between the control and endophyte inoculation (Duncan’s multiple range test *P ≤ 0.05, **P ≤ 0.01).

**Figure 3:**
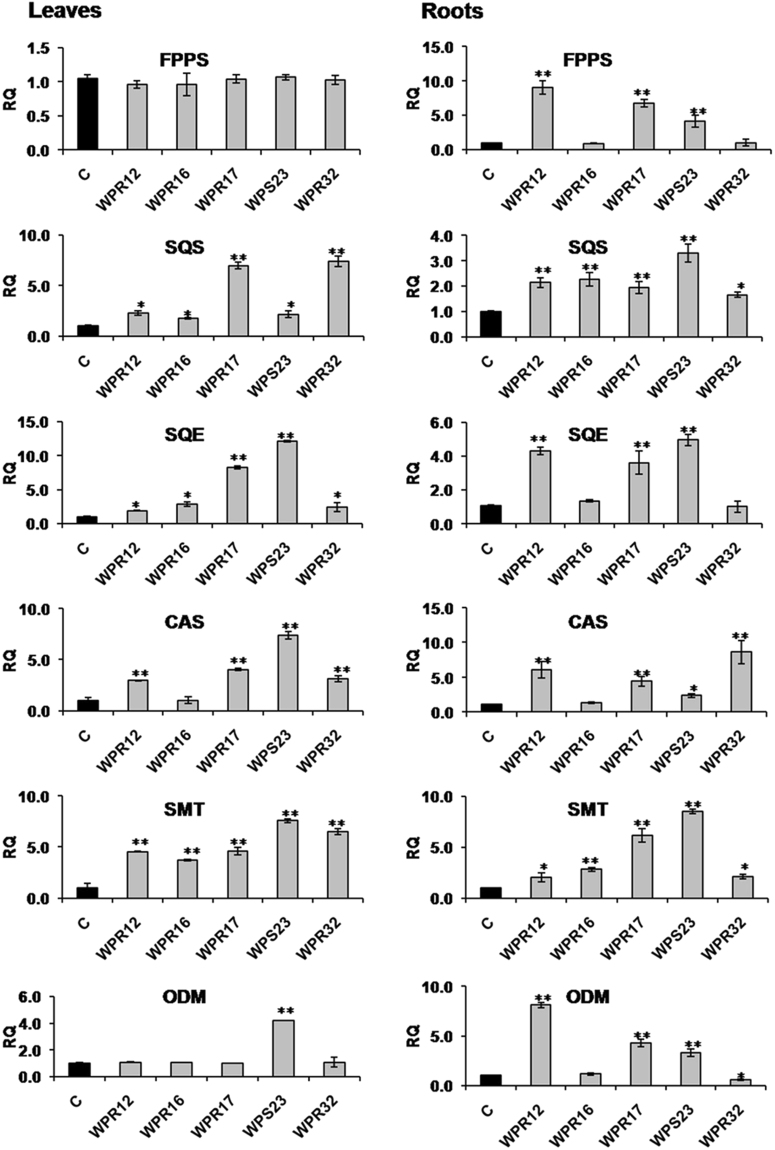
Effect of endophyte inoculation on the expression of genes involved in withanolide biosynthesis. Expression of *FPPS*, *SQS*, *SQE*, *CAS*, *SMT* and *ODM* was analyzed. Results were normalized to actin (reference transcript) and are shown relative to the level in non-inoculated endophyte-free control plants (calibrator). Data are means ± SD (*n* = 3 biological replicates) and *Y*-axis represents relative quantity (RQ). Asterisks indicate significant differences between the control and endophyte inoculation (Duncan’s multiple range test *P ≤ 0.05, **P ≤ 0.01).

To understand the mechanism of the increased withanolide production in leaves and roots of *Withania* plants because of endophyte inoculation, expression of genes involved in withanolide biosynthesis was studied using qRT-PCR. We selected WPR12, WPR16, WPR17, WPS23, and WPR32 inoculated plants to understand the endophyte-mediated modulation of withanolide biosynthesis because these endophytes could substantially enhance biosynthesis of more than one withanolide simultaneously. A total of 12 genes transcripts were quantified in leaves and root tissues. Expression of MVA-pathway gene *HMGR* was found to be upregulated in leaves and roots of the selected endophyte-inoculated plants (Fig. 2). Expression of MEP-pathway gene *DXS* and *DXR* were not affected in leaves of endophyte-inoculated plants (Fig. 2). However, WPR12, WPR17, WPS23 and WPR32 inoculation upregulated the expression of *DXS* in roots. The expression of *DXR* in roots of all the selected endophyte-inoculated plants was upregulated (Fig. 2). In leaves, the expression of *FPPS* remained unaffected in endophyte-inoculated plants (Fig. 3). However, WPR12, WPR17, and WPS23 inoculated plants showed upregulated *FPPS* expression. WPR12, WPR16, WPR17, WPS23 and WPR32 inoculated plants had increased expression of *SQS* in both leaves and root (Fig. 3). All the selected endophyte upregulated the expression of *SQE* in leaves of *Withania* plant (Fig. 3). However, its expression in roots could be upregulated in plants inoculated with WPR12, WPR17 and WPS23. *CAS* expression was upregulated in leaves and roots of WPR12, WPR17, WPS23 and WPR32 inoculated plants whereas *SMT* expression was upregulated in leaves and roots of all the selected endophyte-inoculated plants (Fig. 3). Expression of *ODM* was upregulated in leaves of WPS23 inoculated plants, however, its expression in roots was observed to be higher in WPR12, WPR17 and WPS23 inoculated plants compared to non-inoculated endophyte free control plants (Fig. 3).

**Figure 4:**
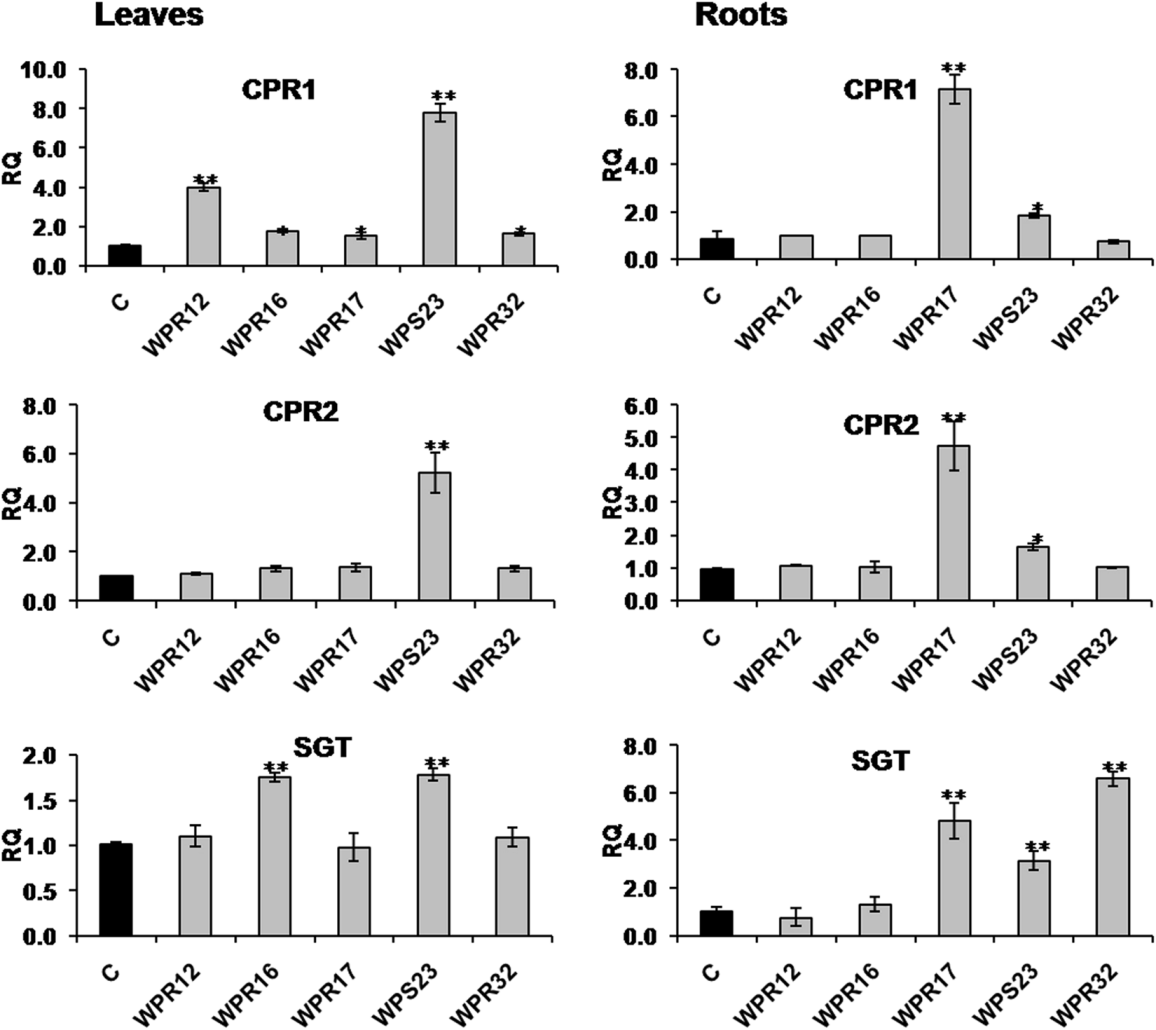
Effect of endophyte inoculation on the expression of genes encoding cytochrome P450 reductases and sterol glucosyltransferases. Expression of cytochrome P450 reductase (*CPR1* and *CPR2*) and sterol glucosyltransferases (*SGT*) was analyzed. Results were normalized to actin (reference transcript) and are shown relative to the level in non-inoculated endophyte-free control plants (calibrator). Data are means ± SD (*n* = 3 biological replicates) and *Y*-axis represents relative quantity (RQ). Asterisks indicate significant differences between the control and endophyte inoculation (Duncan’s multiple range test *P ≤ 0.05, **P ≤ 0.01).

**Figure 5:**
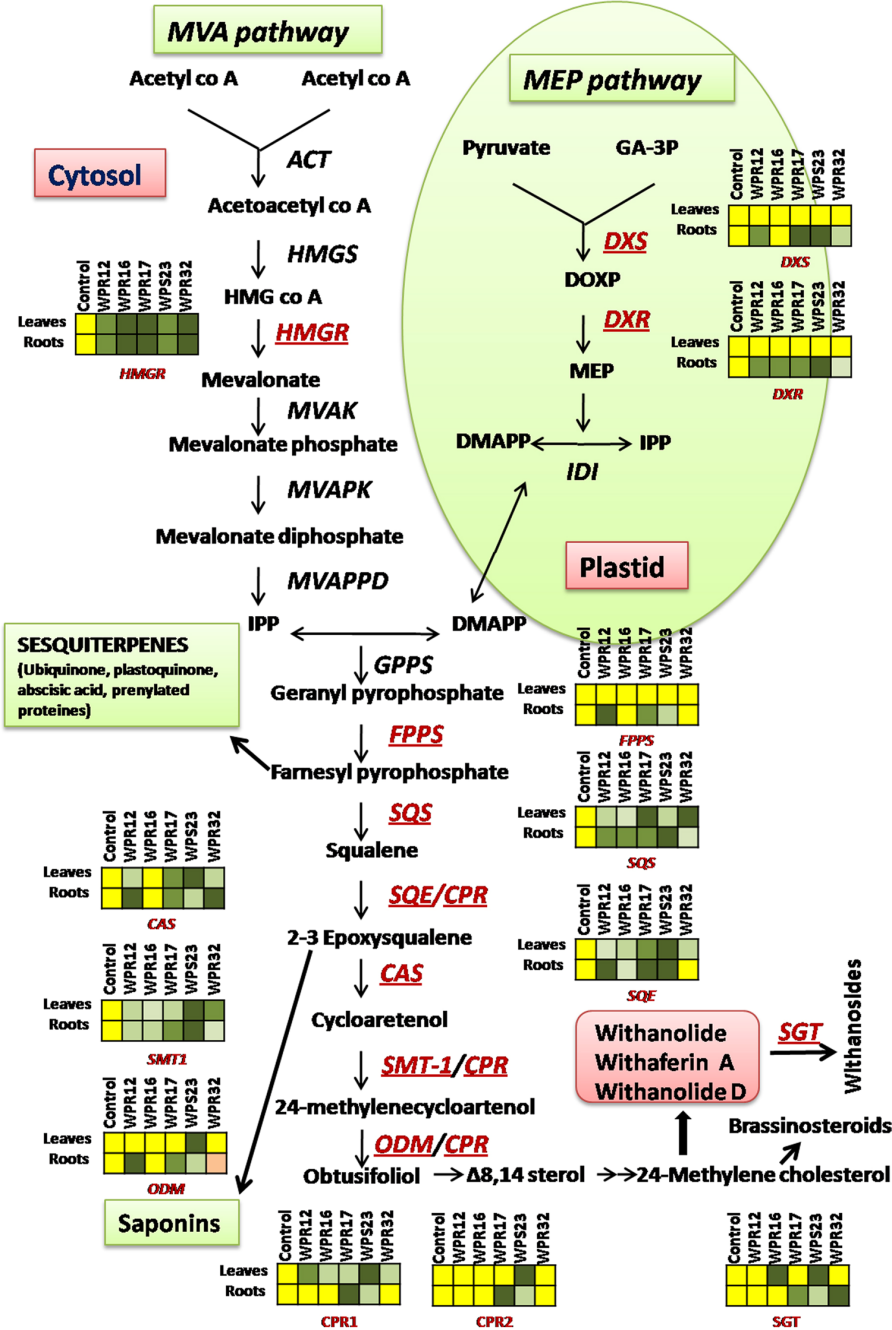
Differential modulation of withanolide biosynthesis by different endophytes. Endophytes isolated from *Withania somnifera* plants modulated the expression of most of the genes of withanolide biosynthetic pathway. Expression of *HMGR* in both leaves and roots was increased by all the selected endophytes. Most of the selected endophyte-inoculation upregulated the expression of *SQS*, *SQE*, *CAS* and *SMT* that could enhance the production of withanolide in leaves and root of *W. somnifera* plants. WPR17 and WPS23 could upregulate all the key genes of withanolide biosynthetic pathway in roots of *W. somnifera* plant. Expression of different genes was presented in square boxes. Green colour of boxes indicate upregulated expression (intensity of green colour shows the level of expression i.e. more green more expression and vice versa), yellow colour shows the level of expression in non-inoculated endophyte free control plants and orange colour shows downregulated expression. *Enzyme abbreviations:* HMGR, 3-hydroxy-3-methylglutaryl-coenzyme A reductase; DXS, 1-Deoxy-D-xylulose-5-phosphate synthase; *DXR*, 1-deoxy-D-xylulose-5-phosphate reductase; *FPPS*, farnesyl diphosphate synthase; *SQS*, squalene synthase; *SQE*, squalene epoxidase; *CAS*, cycloartenol synthase; CPRs (1,2), cytochrome P450 reductase; *SMT*, sterol methyl transferase; *ODM*, obtusifoliol-14 *–*demethylase.

Expression of *CPR1* was found to be upregulated in leaves of all the selected endophyte-inoculated plants (Fig. 4). Leaves of WPS23-inoculated plants showed increased expression of *CPR2* than that of endophyte free control plants (Fig. 4). Expression of *CPR1* and *CPR2* was significantly increased in roots of WPR17, and WPS23 inoculated plants. Expression of *SGT* was upregulated in WPR16, and WPS23 inoculated plant leaves (Fig. 4). WPR17, WPS23 and WPR32 inoculation significantly increased the expression of *SGT* in *Withania* roots. Differential modulation of expression of different genes of withanolide biosynthesis by inoculated endophytes is represented in Fig. 5.

### Effect of endophyte-inoculation on *in planta* IAA content

**Figure 6:**
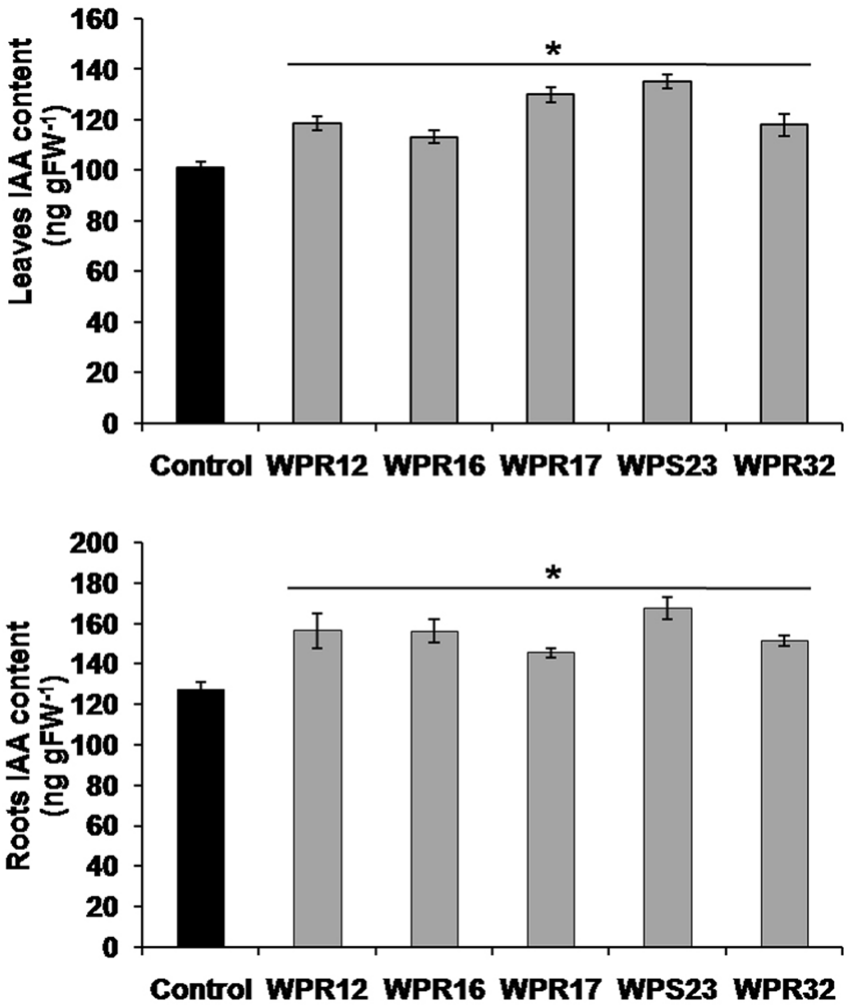
Effect of endophyte-inoculation on indole-3-acetic acid content of leaves and roots of *Withania* plants. Indole-3-acetic acid (IAA) content was estimated in leaves (third leaf from top) and roots of 90 days old endophyte inoculated and non-inoculated endophyte free control *Withania* plants. Each data point is an average of six replicates and the error bars represent standard errors. Asterisks indicate significant differences between the control and endophyte inoculation (Duncan’s multiple range test *P* ≤ 0.05).

Selected endophytes (WPR12, WPR16, WPR17, WPS23 and WPR32) having the potential to enhance *in planta* withanolide production were found to be positive for IAA production (Supplementary Table S4). Therefore, to study the effect of these endophyte-inoculations on IAA level of plants, *in planta* IAA content in roots as well as leaves was quantified. Endophyte inoculation affected the IAA content significantly in both leaves and root tissue (Fig. 6). IAA content was found to be higher in roots than in leaves, and endophyte inoculated plants always had higher IAA content in both leaves and roots than that of non-inoculated endophyte free control plants (Fig. 6).

### Effect of endophyte-inoculation on *in-vitro* grown *W. somnifera* plant

**Figure 7:**
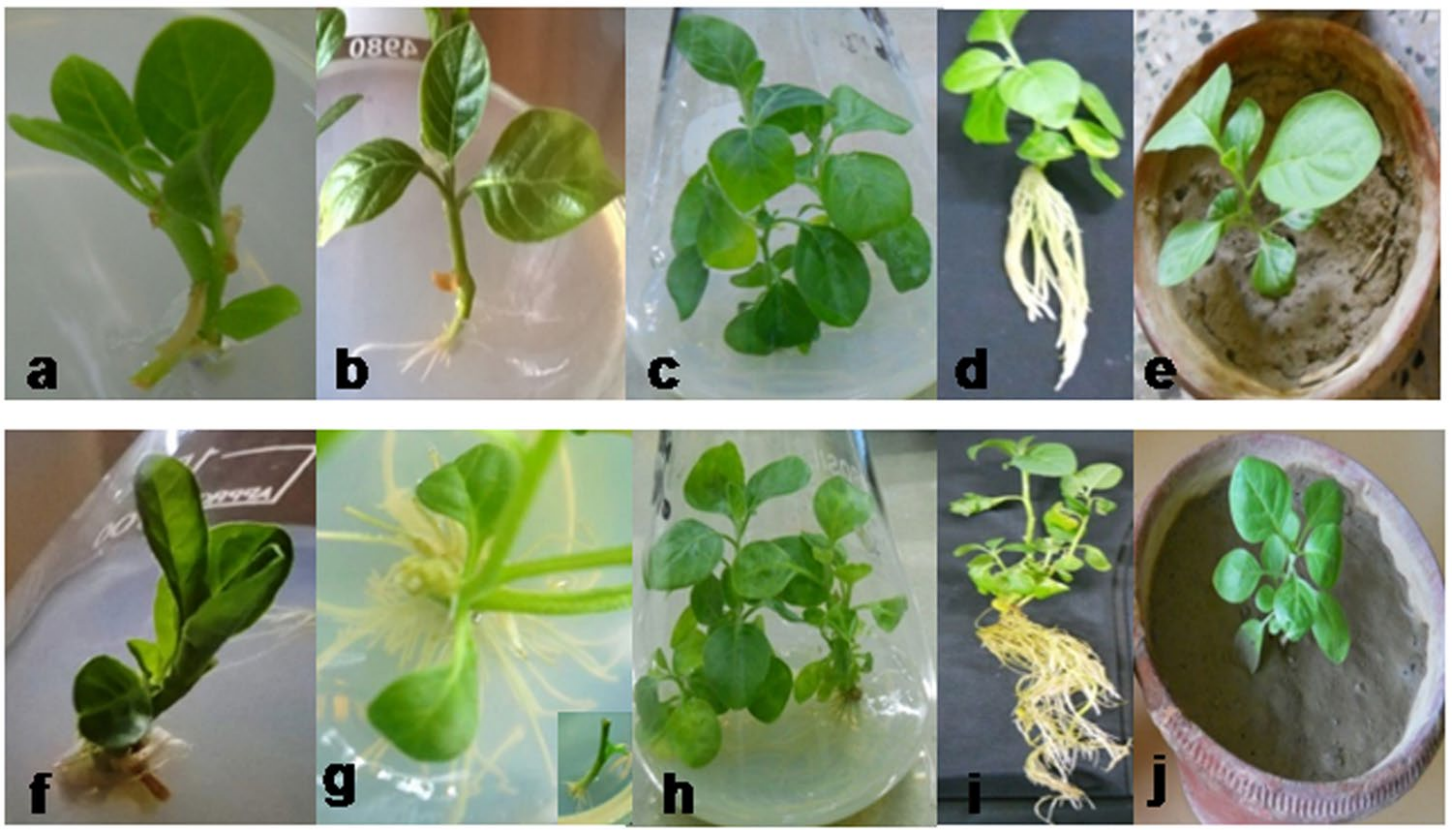
Generation of *in-vitro* grown *Withania somnifera* plant. Establishment of normal plant (**a**) shoot formation in MS medium supplimented with 2 mg L ^−1^ BAP & 0.1 mg L^−1^ NAA; (**b**) rooting initiation on ½ MS medium; (**c**) Complete *in-vitro* establishment of normal plant (**d**) shoot and root after harvesting; (**e**) hardening of normal plant under glass house condition) Establishment of composite plant [(**f**) shoot initiation in MS medium without PGR infected with *A. rhizogenes;* (**g**) transformed root initiation after 15–20 days of infection and root elongation after 35–40 days of inoculation; (**h**) complete *in-vitro* raised composite plant; (**i**) transformed root morphology after harvesting; (**j**) established composite plant under glass house condition].

*In-vitro* plants were developed from nodal explants of *W. somnifera*. Multiple shoot regeneration through axillary bud proliferation of the nodal explants was observed on MS medium containing 2 mg L^−1^ BAP and 0.1 mg L^−1^ NAA, within 3–4 weeks of culture. Rooting was induced on transfer of this plantlets to half strength MS medium without any plant growth regulator (Fig. 7).

Composite plants were raised by inoculating the wild type A4 strain of *A. rhizogenes* to the cut ends of the *in-vitro* raised shoots, where profuse root emergence could be observed after 15–20 days of bacterial inoculation (Fig. 7). These composite plantlets with *A. rhizogenes* induced roots were subsequently transferred to the pre-optimized half strength MS medium for their further establishment and were maintained under the confined environment of glass house for further experimentations with the endophytes (data not shown). PCR analysis showed the presence of the *Rol A*, *B* and *C* genes in the transformed roots of these composite *W. somnifera* plants (Supplementary Fig. S1). The areal parts of these composite plants did not reveal any observable morphological difference with that of the normal plants. However, a striking difference was noticeable in their rooting behavior as the composite plants demonstrated enormous proliferation and elongation of root growth (Fig. 7). The fresh weight of the harvested roots of the composite plants was found to be 4.29 ± 0.29 g plant^−1^ which was 2.66 fold higher than that of the normal plant roots (1.61 ± 0.21 g plant^−1^) (Supplementary Fig. S2).

Subsequent experimentation through inoculation with the three selected endophytes (WPR16, WPR17 and WPS23) to the *in-vitro* raised normal and composite *W. somnifera* plants (which were maintained under the confined environment of glass house) revealed diversified influence on the biosynthetic potentials of both their leaves and roots pertaining to all the three targeted withanolides (i.e., WFA, DWL and WLA). At the outset, the leaves of the composite plants exhibited higher contents of WFA (71.2%), DWL (50%) and WLA (196.7%), compared to that of the normal plants (Table 4). Noticeably, the roots of normal plants reveal the sole presence of WLA with no traces of WFA and DWL at the start while the presence of all the three withanolides could be noted in significant amount in the roots of composite plants (Table 4). Interestingly, the roots of composite plants showed 593% higher WLA content compared to that of the normal plants’ roots without any endophyte treatment.

**Table 4:** Withanolide content in leaves and roots of endophyte-inoculated *in-vitro* grown *Withania somnifera* plants.

	Leaves			Roots		
	%WFA	%DWL	%WLA	%WFA	%DWL	%WLA
**Normal**						
Control	0.59 ± 0.02^c^	0.08 ± 0.01^c^	0.30 ± 0.01^c^	nd	nd	0.057 ± 0.002^d^
WPR16	0.88 ± 0.09^b^	0.08 ± 0.02^c^	0.69 ± 0.03^b^	nd	nd	0.139 ± 0.013^b^
WPR17	1.11 ± 0.06^a^	0.36 ± 0.02^a^	1.09 ± 0.06^a^	nd	nd	0.223 ± 0.015^a^
WPS23	1.15 ± 0.07^a^	0.27 ± 0.01^b^	0.71 ± 0.01^b^	nd	nd	0.096 ± 0.001^c^
**Composite**						
Control	1.01 ± 0.13^c^	0.12 ± 0.007^b^	0.89 ± 0.01^b^	0.082 ± 0.012^d^	0.01 ± 0.002^d^	0.395 ± 0.006^c^
WPR16	0.67 ± 0.12^d^	0.12 ± 0.001^b^	0.48 ± 0.09^c^	0.321 ± 0.018^a^	0.018 ± 0.003^c^	0.638 ± 0.009^a^
WPR17	1.94 ± 0.09^b^	0.16 ± 0.006^a^	1.88 ± 0.05^a^	0.131 ± 0.009^c^	0.027 ± 0.002^b^	0.414 ± 0.004^c^
WPS23	2.3 ± 0.13^a^	0.15 ± 0.007^a^	1.86 ± 0.04^a^	0.153 ± 0.008^b^	0.053 ± 0.002^a^	0.566 ± 0.008^b^

WFA- withaferin A, DWL- 12-deoxy withstramonolide and WLA- withanolide A. Values are the means of six biological replicates ± S.E. Values with different letters are significantly different at *P* ≤ 0.05 (Duncan’s multiple range test). nd- not detected.

Then again, inoculation of the *in-vitro* raised normal plants with the three selected endophytes (WPR16, WPR17 and WPS23) led to the enhancements in the contents of the WFA and WLA in their leaves at 49–95% and 130–263% higher levels respectively as compared to that in the endophyte non-inoculated control plants (Table 4). At the same time, except for WPR16, the other two endophytes (i.e., WPR17 and WPS23) inoculation caused 237–350% stimulation in the DWL content in leaves of normal plants (Table 4). Alternatively, all the three tested endophytes enhanced the WLA content in roots of *in-vitro* raised normal plants by a range of 68–291% as compared to that in the endophyte non-inoculated control plants (Table 4).

On the other hand, inoculation with these two endophytes (WPR17 and WPS23) to the *in-vitro* raised composite *W. somnifera* plants enhanced the foliar contents of all the three withanolides, i.e., WFA, DWL and WLA, by 92–128%, 25–33% and 109–111% respectively, whereas the WPR16 proved ineffective for improvement in DWL content (Table 4). However, every single one of the three tested endophytes stimulated the production of all WFA, DWL and WLA at variable levels in the roots of the composite plants which ranged at 60–291%, 80–430% and 43–61% higher yields respectively, compared to that in the non-inoculated endophyte free control plants’ roots (Table 4).

The overall analysis of the results in terms of the influence of the three tested endophytes on the biosynthetic potentials of the normal and composite plants (in their leaves and roots) pertaining to the three desired withanolides revealed very interesting outcomes which highlights the future applicability of this study. The WPS23 endophyte proved best for enhancing the WFA and WLA contents in the leaves of both the normal and composite plants, but the latter proved most responsive towards this endophyte, resulting in 100% and 162% stimulation in their WFA and WLA productivity respectively compared to that in the former (Table 4). The WPR17 endophyte demonstrated the second optimum efficiency which also boosted the foliar production trend of these two above-stated withanolides (WFA and WLA) in both the control and composite plants, where the latter superseded the former by 74.77% and 72.5% better productivities respectively (Table 4). Noticeably, this WPR17 endophyte had additionally enhanced the foliar content of DWL in the normal plant, which was 125% higher compared to that in the leaves of the treated composite plants (Table 4). On the other hand, the potential of the roots of the composite plants towards the synthesis of all the three desired withanolides in the background of their total absence in the roots of the normal plants, needs special mention, which showed an up-ward augmentation by the inoculation with all the three endophytes with observable variability (Table 4).

**Figure 8:**
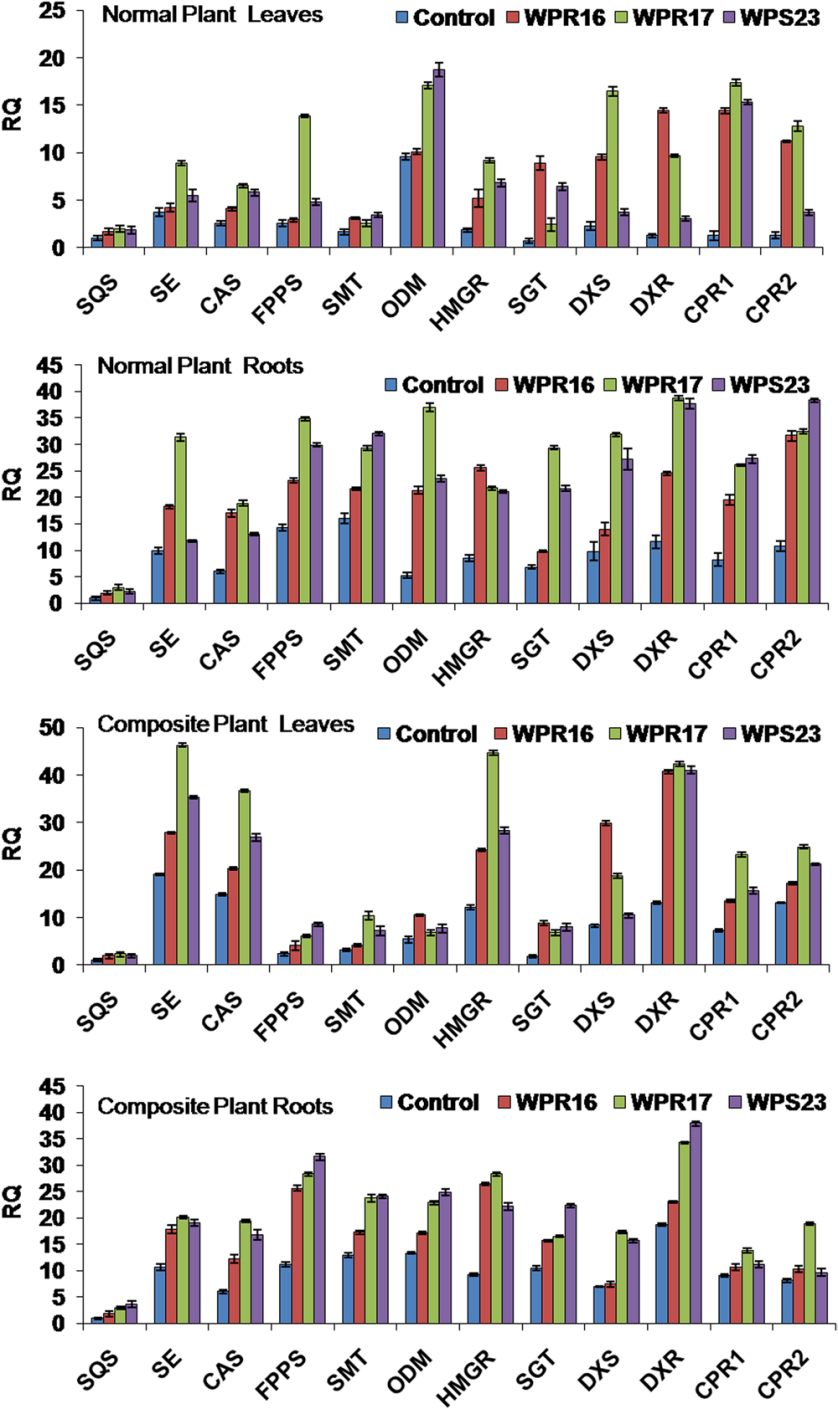
Effect of endophyte inoculation on the expression of withanolide biosynthesis genes in *in-vitro* grown normal and composite *Withania somnifera* plant. Expression of different withanolide biosynthesis genes was analyzed. Results were normalized to actin (reference transcript) and are shown relative to the level in non-inoculated endophyte-free control plants (calibrator). Data are means ± SD (*n* = 3 biological replicates) and *Y*-axis represents relative quantity (RQ).

The expression of different genes involved in withanolide biosynthesis was studied by qRT-PCR in *in-vitro* grown *W. somnifera* plants inoculated individually with selected endophytes (WPL16, WPL17, WPS23). Expression of *SQS*, *SE*, *CAS*, *FPPS*, *SMT*, *ODM*, *HMGR*, *SGT*, *DXS*, *DXR*, *CPR1* and *CPR2* genes were quantified in leaves as well as in roots of *in-vitro* grown normal and composite plants. Expression of *CAS*, *SMT*, *HMGR*, *SGT*, *DXS*, *DXR*, *CPR1* and *CPR2* genes was increased in leaves of endophyte inoculated *in-vitro* normal plants compared to non-inoculated endophyte free control plants, while expression of *SQS*, *SE*, *FPPS*, and *ODM* was upregulated by WPL17-, WPS23-inoculation (Fig. 8). Expression of all studied genes in roots of *in-vitro* normal plants was increased by selected endophyte inoculation (Fig. 8). Similarly, leaves and roots of endophytes inoculated *in-vitro* composite plants also had increased expression of studied pathway genes compared to non-inoculated endophyte-free control plants (Fig. 8).

## Discussion

Endophytes have promising potential for sustainable agriculture as they can promote plant growth and confer tolerance to plant from environmental stresses^31–35^. They are also the source of therapeutically important novel chemicals^36–38^. Earlier work from our laboratory has indicated that some endophytes may enhance the secondary metabolite production of the medicinal plants^39,40,42,43^. We have shown that medicinal plant opium poppy harbour numerous endophytes residing in different parts of the plants and play a role in a tissue-specific manner^39^. Endophytes associated with opium leaves were involved in improving photosynthetic efficiency of the plant while the endophytes associated with capsule (major site for secondary metabolites production) were involved in improving secondary metabolites specifically benzylisoquinoline alkaloid production^39^. No such relation was observed in *Withania* plants as far as shoot growth is concerned as the endophytes isolated both from leaf and root could enhance the shoot growth. However, endophytes isolated from roots could significantly enhance the root growth, supporting our earlier study suggesting their tissue specific roles^39^. Two fungal endophytes *Curvularia* sp. and *Choanephora infundibulifera* isolated from *Catharanthus roseus* enhanced *in planta* production of vindoline by regulating the expression of key structural and regulatory genes of terpenoid indole alkaloid biosynthesis^40^. In the present study, we demonstrated an important role of endophytes in improving plant yield and secondary metabolite production in medicinally important *W. somnifera* plant. We could isolate and identify a total of 29 bacterial endophytes and 11 fungal endophytes. The role of these isolated endophytes was studied by inoculating them in endophyte-free plants and compared with the non-inoculated endophyte-free control plant. It was observed that few isolated fungal endophytes could improve the photosynthetic efficiency of *W. somnifera* plant by improving the content of photosynthetic pigments (Chlorophyll and carotenoids), net CO_2_ assimilation rate, transpiration rate and stomatal conductance. Improved photosynthetic efficiency due to endophyte inoculation resulted in increased biomass of *Withania* plant that was evident from the higher shoot and root biomass. As both leaves and roots are the economic parts, used for the extraction of withanolides, therefore, these fungal endophytes can be used for the substantial improvement of biomass. Improved growth in endophyte-inoculated *Withania* plants may be due to indole acetic acid production and phosphate solubilization activity of endophytes.

Withanolides are the major secondary metabolites responsible for pharmacological properties of different parts of *W. somnifera* plant. Inoculation with endophytes modulated the content of withanolides in leaves and roots of *W. somnifera* plant. Surprisingly, some endophytes induced the synthesis of withaferin A in roots (0.010–0.043%; Table 3) which is abundantly synthesized in leaves^17–19^ and totally absent^16^ or present in traces (0.0005–0.0007%) in roots of some varieties^20^ of *W. somnifera*. This clearly shows a strong involvement of endophytes in modulating the biosynthetic pathway. Expression study of different genes involved in withanolide biosynthesis revealed the possible mechanism associated with endophyte mediated changes in withanolide biosynthesis. We observed that endophyte inoculation modulated the expression of genes of withanolide biosynthesis. Withanolides biosynthesis involves both MVA and MEP pathways which regulate the flux of the isoprene units (isopentenylnpyrophosphate; IPP and dimethylallyl pyrophosphate; DMAPP) for the synthesis of intermediates of withanolide biosynthesis^47^. Endophytes inoculation upregulated the expression of *HMGR* (both in leaves and roots), involved in the biosynthesis of isopentenylnpyrophosphate (IPP) via MVA pathway which is the predominant pathway for biosynthesis of withanolides^48^, and this might be a key reason for the higher production of withanolides in endophyte-inoculated plants. The expression of *DXR* and *DXS*, involved in Methylerythritol 4-phosphate (MEP) pathway, was not affected in leaves of the endophyte-inoculated plant which might be due to the existence of distinct/specific transcription regulatory mechanism for genes encoding for enzymes involved in MEP pathway^49^. However, endophytes could upregulate the expression of *DXS* and *DXR* in roots. It might be due to the constant association of these endophytes with roots (their site of isolation) that could specifically modulate their expression in roots. The expression of *DXS*, *DXR* and *FPPS* remained unaffected in the leaves of endophyte inoculated plants, however, their expression was upregulated in roots and this might be a possible reason for induction of withaferin A synthesis in roots. This also indicates the importance of MEP-pathway genes *DXS* and *DXR* in withaferin A biosynthesis. Plant growth promoting activity such as IAA production and nitrogen fixation ability of these root associated endophytes (WPR12, WPR16, WPR17, WPS23 and WPR32) might also facilitate production of withaferin A in roots. Selected endophyte inoculation could increase the *in planta* content of IAA in leaves and roots that could enhance withanolide biosynthesis. Improvement of withaferin A production by application of nitrogen significantly upregulating structural and regulatory genes of withanolide biosynthesis also supports our present observation^50^. The previous report demonstrated that IAA favours the biosynthesis of withaferin A in *in-vitro* root cultures of *W. somnifera*^51^ consolidates the role of IAA producing endophytes in enhancing the content of withaferin A in the present study. Expression of *SQS*, encoding squalene synthase catalysing the condensation of farnesyl pyrophosphate (FPP) to produce squalene (a key intermediate for biosynthesis of various triterpenoids) was found to be upregulated in leaves and roots of all the selected endophyte-inoculated *Withania* plant. Overexpression of *SQS* in *W. somnifera* resulted in increased production of withanolides^23^ and virus-induced gene silencing (VIGS) of *SQS* in *W. somnifera* led to the reduction in withanolide synthesis confirmed the crucial role of *SQS* in withanolide biosynthesis^52^. Overexpression of *SQS* in cell suspension culture of *W. somnifera* substantially enhanced (2.5 fold as compared to non-transformed cell cultures) withanolide production and also started withaferin A biosynthesis which was absent in non-transformed cell cultures^29^. Therefore, upregulated expression of *SQS* (up to 3.3 folds) in endophyte inoculated plants might induce the synthesis of withanolides especially withaferin A in roots which was clearly visible in WPR 16 and WPS 23 inoculated plants. Endophyte-inoculated plants had upregulated expression of *SQE* which encodes squalene epoxidase which is a rate limiting enzyme in the withanolide biosynthetic pathway^53,54^. Cascading influence of *SQE* on the upregulation of downstream genes and its genetic manipulation in *W. somnifera* plant suggest a promising way for the production of therapeutically important triterpenoid molecules^55,56^. Therefore, higher expression of *SQE* (up to 4.9 folds in roots and 12.1 folds in leaves) in the endophyte-inoculated plants might enhance the expression of downstream genes that could have resulted in higher withanolide production both in roots and leaves.

Endophyte-inoculation could upregulate the expression of *CAS* which forms cycloartenol (the precursor for withanolide biosynthesis) and acts at metabolic branching point for synthesis of withanolides and various triterpenes such as saponins (Fig. 5). Previous reports show that *CAS* overexpression and silencing resulted in an increase and decrease of withanolide content respectively^24^. In endophyte-inoculated plants, increased expression of *CAS*, *SMT* and *ODM* were correlated with enhanced withanolides production. Few endophytes modulated the expression of *CPRs* which encode cytochrome P450 reductase. P450s are involved in the primary and secondary metabolism of a plant by catalysing various reaction such as hydroxylations, epoxidations, sulfoxidations, dealkylations, peroxidations, reductive dehalogenations and isomerisation reactions^57,58^. Endophytes also modulated the expression of *SGT* (sterol glucosyltransferases) responsible for glyco-transformations of steroids.

Figure 5 shows differential regulation of withanolide biosynthetic pathway by inoculated endophytes. All the selected endophyte-inoculations upregulated the expression of *HMGR* in both leaf and root tissues, indicating that it may be a promising candidate gene that can be used for increasing *in planta* withanolide production. Previously, *HMGR* as rate limiting enzyme of isoprenoids biosynthesis of eukaryotes has also been suggested^59^. *Piriformospora indica* elicitation increased withaferin A biosynthesis by upregulating the regulatory genes of MEP, MVA and withanolide biosynthetic pathway and showed the highest expression of *HMGR* in cell suspension cultures of *Withania somnifera*^60^. Most of the endophyte-inoculation increased the expression of *SQS*, *SQE*, *CAS* and *SMT* that could enhance the production of withanolides in both leaves and root tissues. The expression of *DXS*, *DXR* and *FPPS* which was not affected in leaves, indicates the presence of different regulatory components/factors that were not targeted by endophytes. WPR17 and WPS23-inoculation upregulated the expression of all the key genes of withanolide biosynthesis in roots. Therefore, these are the most promising candidates that can be used for the improvement of *in planta* withanolide production.

As roots of the *W. somnifera* plants are the major source of withanolides and used for herbal therapeutics, therefore, in the present study we have evaluated the effect of the selected endophytes on *in-vitro* grown composite plants having higher root biomass than that of the normal plants. The utilization of composite plants had earlier been reported as a successful alternative approach for the functional characterization of any targeted gene(s) in the host plants, where the transformation and regeneration are tedious and lengthy^45^. This technology has also been found competent for several other studies such as nutrient and hormone uptake, interactions with root nodulating bacteria and mycorrhizal symbiotic association^46^. The present study has first time explored the potentials of the composite plants with respect to evaluating the influence of external applications of targeted endophytes with the overall intention of amplifying the host-endophyte interaction interphase (to best of our knowledge). Here, we developed the composite plant of *W. somnifera* to evaluate the potentials of such transformed roots towards the biosynthesis of secondary metabolites with and without treatment of selected endophytes isolated from *W. somnifera* and found to enhance the secondary metabolite production.

Normal plants were regenerated *in-vitro* from nodal explants, and composite plants were generated by inoculating wild-type *A. rhizogenes* A4 strain to the cut ends of *in-vitro* raised shoots resulting in the production of profuse rooting. Successful integration of *rol* gene in the genome of normal plants could result in the development of composite plants having more roots than the normal plants. Shoot and root regeneration in nodal explants was found to be similar as reported previously^61–66^. Generation of composite plants also enhanced the biosynthesis of all the measured withanolide (WFA, DWL and WLA) in both the leaf and root tissues. Furthermore, in general the inoculation with selected endophytes (WPL16, WPL17 and WPS23) enhanced the content of all the measured withanolide (WFA, DWL and WLA) in the leaves and roots of the composite plants, which is due to the upregulation of the expression of key genes of withanolide biosynthesis as observed in the case of *in-vivo* normal plants. qRT-PCR study showed the upregulation of *HMGR* in both the leaves and root tissues of normal *in-vivo* as well as *in-vitro* grown normal and composite plants. This observation strongly indicates that *HMGR* could be the key gene that can be considered as the target for enhancing the withanolide content in *Withania* for genetic manipulation/transgenic considerations. Reduction in the content of WFA in leaves of WPR16 inoculated composite plants, DWL in WPR17 and WPS23 inoculated plants and WLA in WPR16 inoculated plants indicates endophyte mediated change of flux of withanolide biosynthesis from leaves to roots as these alkaloids accumulated in increased amounts in their root tissue. Similarly presence of WFA and DWL in roots of composite plant (which was not detected in normal plants) in both endophyte inoculated and non-inoculated plants also indicates the change of flux of withanolide biosynthesis from leaves to roots, which might have resulted due to the Ri T-DNA mediated changed nature of the composite plant.

To summarize, results of the present study suggest that the medicinal plant *W. somnifera* harbours various bacterial and fungal endophytes associated with leaves, roots and seeds. These endophytes may play an important role in the biosynthesis of important secondary metabolites and also have the potential to improve plant shoot and root biomass by improving the photosynthetic pigments, photosynthesis rate, transpiration rate and stomatal conductance through growth promotion activities such as IAA production and phosphate solubilization. We also demonstrated that *W. somnifera* have endophytes which have the ability to modulate the withanolide biosynthesis in the leaves and roots of *W. somnifera* by modulating the expression of key genes of withanolide biosynthetic pathway. The presence of few IAA-producing and nitrogen-fixing root-associated endophytes that can induce the production of leaf alkaloids (withaferin A) in roots by upregulating the expression of withanolide biosynthesis genes especially MEP pathway genes (*DXS* and *DXR*) as promising candidates for enhancing the withaferin A biosynthesis in *Withania* root is also suggested. Application of these endophytes in *Withania* agriculture will benefit growers to harvest higher amounts of both withanolide A and withaferin A from roots. Although the mechanism associated with endophyte-mediated modulation of host plant metabolism is really difficult to unravel, their possible role in promoting plant growth and yields, and pharmaceutically important withanolide biosynthesis cannot be ignored. Formulation and testing of microbial consortia consisted of endophytes enhancing plant biomass and endophytes enhancing withanolide content may empower host plant to produce higher biomass coupled with more withanolide content.

## Methods

### Plant material and growth conditions

Seeds of *Withania somnifera* genotype Poshita were obtained from the National Gene Bank for Medicinal and Aromatic Plants, CSIR-Central Institute of Medicinal and Aromatic Plants (CSIR-CIMAP), Lucknow, India and grown in a greenhouse in natural photoperiod of 16 h day/8 h night cycle under natural light intensity at 25 °C ± 2 °C. *Withania* plants were grown in earthen pots (22 cm top diameter × 12 cm bottom diameter × 17 cm height and 3.7 L volume) filled with potting mixture (3.5 kg) consisted of autoclaved soil and vermicompost (2:1, v/v) and watered with sterile water.

### Isolation and identification of endophytes

Endophytes were isolated from healthy green leaves, root and seeds of field-grown *Withania somnifera* genotype Poshita plant following the procedure described previously^39,40^. Isolation of endophytes was done in triplicate. Each replicate of leaves (1 g) and roots (1 g) was collected from three individual plants. Approximately 100 mg seeds was used in triplicate for endophyte isolation. Initially, surface sterilization of plant tissues was done by placing tissues in 1% sodium hypochlorite solution for 10 min and then washed (five times) in 0.02 M sterile potassium phosphate buffer (pH 7.0). Sterility check of tissues was performed by adding 100 µl of an aliquot of the final buffer wash into 5 ml nutrient broth (g l^−1^; 5 g peptone, 2 g beef extract) and incubated in an incubator shaker (200 rpm at 28 °C) for 72 h. Samples were discarded if the growth was detected in the sterility check samples. Surface sterilized tissues were then homogenised in a sterile pestle and mortar with sterile distilled water (10 ml). Homogenate was serially diluted (10^−1^, 10^−2^ and 10^−3^) in sterile distilled water and plated on nutrient agar (NA) (g L^−1^; 5 g peptone, 2 g beef extract, 20 g agar, pH 5.0) and potato dextrose agar (PDA) (g L^−1^; 200 g potato infusion, 20 g dextrose, 15 g agar, pH 5.6) plates in triplicates. Bacterial endophytes were selected on NA plates, incubated at 28 °C for 24–72 h and fungal endophytes were selected on PDA plates incubated at 28 °C for 10 days. A representative of each endophyte (as distinct from their colony morphology) was transferred to a fresh plate and pure culture was established. Identification of bacterial and fungal endophytes was performed by 16 S rRNA and internal transcribed spacer (ITS) sequencing respectively. Amplification and sequencing of 16 S rRNA and ITS fragments were performed following the procedure described earlier^39,40^. Similarities in nucleotide sequences were determined by using NCBI BLAST, version 2.0. Partial sequence data of 16 S rRNA and ITS fragment have been submitted to the NCBI GenBank, and accession numbers were obtained (Table 1).

### Inoculum production for endophytes

Bacteria were grown in nutrient broth to the mid-log phase and then pelleted by centrifugation at 4000 rpm for 10 min at 4 °C temperature. The pellet was washed and resuspended (1 × 10^8^ CFU mL^−1^) in phosphate buffer saline (PBS) (g L^−1^; 0.24 g potassium dihydrogen phosphate, 1.44 g disodium hydrogen phosphate, 8 g sodium chloride, 0.2 g potassium chloride, pH 7.4). For fungi inoculum preparation, the individual fungus was grown in potato dextrose broth for one week at 28 °C. Numbers of conidia/spore were counted on microscope and dilution (1 × 10^8^ spores/conidia mL^−1^) was made in PBS.

### Treatment of *Withania* plants with endophytes

Endophyte-free *Withania* plants were used to study the effects of treatment with isolated endophytes. Endophytes-free *Withania* plants were generated following previously established procedure^39,40^. In brief, *Withania* seeds were extensively washed in water and then incubated in Bavistin (a fungicide containing carbendazim 50% W.P., BASF India Limited) and K-Cycline (a bactericide containing streptomycin sulphate 90% w/w and tetracycline hydrochloride 10% w/w, Karnataka Antibiotics & Pharmaceuticals Ltd. Bangalore, India) solution at 28 °C temperature, 120 rpm shaking for 72 h and then washed extensively in sterile water and homogenized in a sterile pestle and mortar with sterile PBS. Homogenate was plated on NA and PDA and incubated at 28 °C for 10 days. No microbes were obtained on incubated plates. Therefore, these *W. somnifera* seeds were used as endophyte free seeds and used for growing endophyte-free nursery. The nursery was grown in earthen pots (30 cm top diameter × 20 cm bottom diameter × 7 cm height) filled with of autoclaved potting mixture (3.0 kg) consisted of soil and vermicompost (2:1 v/v) and watered with sterile water. These pots were grown under greenhouse condition (natural photoperiod and light intensity at 25 °C ± 2 °C) for one month, and grown seedlings were re-confirmed for their endophyte-free status. These seedlings were uprooted delicately to minimize the damage to their roots and inoculated with isolated endophytes. For endophyte inoculation, roots of seedlings were dipped in individual endophyte suspension (1 × 10^8^ CFU mL^−1^ for bacteria and 1 × 10^8^ spore/conidia mL^−1^ for fungus) prepared in PBS for 3 h. These endophyte-inoculated seedlings were re-planted in pots (22 cm top diameter × 12 cm bottom diameter × 17 cm height) filled with autoclaved soil and vermicompost mixture (2:1 v/v) and grown under greenhouse conditions. The roots of non-inoculated endophyte-free control were dipped in PBS for the same duration. Plants were watered with sterile water as and when required. In all experimental analyses, endophyte-inoculated plants were compared with the non-inoculated endophyte-free control plants that developed from endophyte-free *Withania* seeds. Inoculation (10 mL pot^−1^ containing 1 × 10^8^ CFU mL^−1^ bacterial cell or 1 × 10^8^ fungal spore/conidia mL^−1^) with individual endophyte was again repeated after 15-days of the first inoculation to maintain the presence of an adequate number of inoculated endophytes in the soil. Presence of inoculated endophytes in *Withania* plants was confirmed by re-isolating the endophytes from tissues of endophyte-inoculated plants before carrying out further experimental analyses.

Sampling for all analyses was carried out at the same stage (90 d which is an intermediate stage neither too young nor too old) and position of leaf (third leaf from top) for minimizing the variations dependent on developmental stage of plant and other variables.

### Photosynthetic pigments and photosynthesis parameters analysis

Photosynthetic pigments (Chlorophyll and carotenoids) and photosynthetic efficiency of third leaves of 90-days old *Withania* plants (n = 3) were determined. Pigments were extracted in chilled 100% methanol, and the content of chlorophyll and carotenoids was measured following to previously established procedure^67^. Photosynthesis parameters (net CO_2_ assimilation, transpiration rate and stomatal conductance) were measured in the attached leaves using a portable photosynthesis system (CIRAS-3, PP Systems, USA). For photosynthesis measurement, leaf was pre-exposed for 15 min at 400 µmol photons m^−2^ s^−2^ light, 400 ppm CO_2_ and 25 °C temperature.

### Establishment of *in-vitro* grown normal plants of *W. somnifera*

*W. somnifera* cv. Poshita, cultivated in the field of CSIR-CIMAP, Lucknow were used as explants source for establishment of *in-vitro* culture. *In-vitro* culture of *W. somnifera* was established using nodal explants according to the previously published protocol^68^. In short, the nodal explants were initially washed with 10% (v/v) Tween-20 for 5 min followed by washing with running tap water. Surface sterilization of explants was done with 0.1% HgCl_2_ and then inoculated on MS medium^69^ supplemented with 0.1 mg L^−1^ NAA and 2 mg L^−1^ BAP for multiple shooting and further transferred in half strength of MS medium without any supplementation of plant growth regulator for the establishment of the complete plant. After the successful establishment of *in-vitro* culture (with roots and shoots) they were transferred to sterilized soil, hardened and acclimatized under confined glasshouse condition (natural photoperiod and light intensity at 25 °C ± 2 °C and 80% relative humidity)

### Establishment of *in-vitro* grown composite plants of *W. somnifera*

The transformation experiments for the development of composite plants were carried out following previously published protocol^62^ with slight modification utilizing the wild type of A4 strain of *A. rhizogenes* (a kind gift from Prof. D. Tepfer, INRA, Versailles Cedex, France). In this study, the needle-pricking method was applied at the cut end of nodal explant by inoculation of over-night grown bacterial suspension (O.D 600 = 1.0). After 2–3 days of co-cultivation of explants on half strength MS medium with the *Agrobacterium*, the explants were transferred to ½ MS containing 100 mg L^−1^ of Cefotaxime (Alkem, India). Similarly, the complete composite plants (with normal shoots & transformed roots) were transferred to sterilized soil, hardened and acclimatized under glasshouse condition.

Inoculation of the selected endophytes (WPL16, WPL17, WPS23) was done after the transfer of *invitro* grown plants (normal and composite) to sterilized soil. The roots of *invitro* grown plants were dipped in individual endophyte suspension (1 × 10^8^ CFU mL^−1^) prepared in PBS for 3 h and then planted in the pot filled with autoclaved soil. For control plants, only PBS (without endophyte) was used. Plants were grown under confined glasshouse condition.

### Confirmation for *Rol* genes in transformed roots of composite plants

DNA was extracted from the *in-vitro* raised transformed roots of composite plants (CPR) and normal plant root (NPR) according to the protocol described previously^70^. *Rol A*, *B* and *C* genes were PCR amplified using the *rol* specific primer for *Rol A* (Forward: 5′-GGAATTAGCCGGACTAAACG-3′ and Reverse 5′-CCGGCGTGGAAATGAATCG-3′), *Rol B* (Forward: 5′-ATGGATCCCAAATTGCTATTCC-3′ and Reverse: 5′-GTTTACTGCAGCAGCAGCAGGCTTCATG-3′) *Rol C* (Forward: 5′-ATGGCTGA AGACGACCTGTGT-3′ and Reverse: 5′-CCGATTGCAAACTTGCACTC-3′) were used. The PCR conditions were followed as described previously^70^.

### Measurement of plant biomass

Shoot and root biomass of 90-days old *W. somnifera* plant (n = 6) were measured. Entire shoots (aboveground plant parts) and entire roots (belowground plant parts), were harvested and completely dried at 70 °C for 5 d, and their dry weight was measured.

### Analysis of withanolide

Withanolide A (WLA), withaferin A (WFA) and dideoxywithanoliside (DWL) content was estimated in leaves and roots of *Withania* plant by HPLC. Leaves (third from top) and roots of 90-days old *Withania* plant were taken for alkaloid extraction. In the case of *in-vitro* grown normal and composite *Withania* plant leaves and roots samples were taken at 60 d stage after glass house acclimatization. Tissues were dried and ground to fine powder. 100 mg of powdered samples were extracted with 2 mL warm (50 °C) methanol for 4 h. The extraction process was repeated three times. Methanol extract was filtered through Whatman No. 1 filter paper and concentrated by drying and re-dissolved in methanol for HPLC analysis. The withanolide content was estimated by HPLC equipment (Shimadzu, Japan) containing Spherisorbs® C18 (250 mm × 4.6 mm i.d.) 10 μm particle size ODS2 column (Waters, Milford, MA), pumps (LC-10AT), an auto-injector (SIL-10AD) and PDA (SPD-M10A) detector^71,72^. Mobile phase composition was 40:60 (v/v) mixture of acetonitrile: trifluoroacetic acid [0.1% (v/v) in water] with the flow rate of 1.0 mL min^−1^ and detector wavelength of 220 nm was used throughout the analysis. All standard withanolides were procured from ChromaDex (Irvine, CA) and all solvents used for analysis were of HPLC grade. The specificity of the measured compounds (WLA, WFA and DWL) was established through peak purity analysis using a PDA detector, matching UV-Vis spectra and spiking with reference compound of known concentration (Supplementary Fig. S3).

### Quantitative real time-PCR (qRT-PCR) analysis of withanolide biosynthesis genes

The qRT-PCR analysis was performed in third leaves (from top), and roots of 90-days old non-inoculated endophyte free control and endophytes inoculated *Withania* plant. In the case of *in-vitro* grown normal and composite *Withania* plants leaves and roots samples were taken at the 60-d stage after glass house acclimatization. Total RNA was isolated using TRI-reagent (Sigma-Aldrich). Concentration of RNA was determined using NanoDrop 1000 spectrophotometer (Thermo Fisher Scientific). RNase-free enzyme DNase I (Thermo Scientific) was used to eliminate genomic-DNA contamination. First-strand cDNA was synthesized using RevertAid First Strand cDNA Synthesis Kit (Thermo Scientific) following manufacturer’s protocol. Transcripts of a total of 12 genes involved in withanolide biosynthesis were quantified. qRT-PCR was performed using SYBR-Green I chemistry on triplicate technical replicates of triplicate biological samples. Primers used for qRT-PCR analysis are described in Supplementary Table S5. PCR mixtures consisted of 300 nM of both forward and reverse primers, 5 μL SYBR Premix Ex Taq (TAKARA BIO INC.), 0.2 μL ROX Reference Dye and 1 µL of 10 times diluted cDNA synthesis reaction in a reaction volume of 10 μL. qRT-PCR condition was an initial denaturation at 95 °C for 10 min; 40 cycles of denaturation at 95 °C for 15 s and annealing/extension at 60 °C for 1 min. Fluorescent signal intensities were recorded and analysed on an Applied Biosystems StepOnePlus^TM^ Real-Time PCR System. Melt-curve analysis using the dissociation method (Applied Biosystems) was included to verify the specificity of RT-qPCR. The actin of *W. somnifera* was used as reference transcript. Non-inoculated endophyte free control plants were used as calibrator, and the relative quantification 2^−∆∆Ct^ method was used^73,74^.

### Screening for growth promotion activity

The phosphate utilization, IAA production and nitrate reduction test was performed following previously established method^75–78^.

### Indole-3-acetic acid measurement

IAA content was estimated using the Phytodetek-IAA Immunoassay kit (Agdia, Elkhart, IN) as described previously^79^. Leaves and root samples were frozen in liquid nitrogen and ground to fine powder. 0.5 g of the powdered tissue was suspended in 5 ml of extraction solution containing 80% methanol, 0.5 g L^−1^ citric acid monohydrate and 100 mg L^−1^ butylated hydroxy toluene, and stirred overnight at 4 °C in the dark. The solution was centrifuged at 1000 g for 20 min at 4 °C and then the supernatant was dried under vacuum. The dried residue was dissolved with 100% methanol (100 µl) and 900 µl of tris-buffered saline (pH 7.8). The IAA concentration in the filtrate was determined using the Phytodetek-IAA Immunoassay kit (Agdia, Elkhart, IN) as per the manufacturer’s instructions.

### Statistical analysis

Statistical analysis of data was done by applying ANOVA, suitable to completely randomised design (CRD), using the software ASSISTAT Version 7.7 beta. Significant differences among different treatments were carried out using Duncan’s multiple range tests (DMRTs) at a significance level of P ≤ 0.05. For measurement of chlorophyll, carotenoids, net CO_2_ assimilation, stomatal conductance, transpiration rate, plant shoot and root biomass, withanolide and IAA content six replicates were used, and for expression analysis, three biological replicates for each treatment were used. Three independent experiments were performed for three consecutive years under greenhouse condition and similar results were obtained.

## Electronic supplementary material

Supplementary Information
