# Supplementary material for: Endophytes of Withania somnifera modulate in planta content and the site of withanolide biosynthesis
Source: Sci Rep. 2018 Apr 3;8:5450. doi: 10.1038/s41598-018-23716-5 (PMC5882813; doi:10.1038/s41598-018-23716-5)
Supplement: Supplementary file 1 — Supplementary Information [file 41598_2018_23716_MOESM1_ESM.pdf]

Supplementary Material

Endophytes of *Withania somnifera* modulate *in planta* content and the site of withanolide biosynthesis

Shiv S Pandey, Sucheta Singh, Harshita Pandey, Madhumita Srivastava, Tania Ray, Sumit Soni, Alok Pandey, Karuna Shanker, CS Vivek Babu, Suchitra Banerjee, MM Gupta, Alok Kalra

Supplementary Table S1 Effect of inoculation with isolated bacterial endophytes on physiological parameters of *Withania somnifera* plants

Treatment	Chlorophyll (mg gFW^{-1})	Carotenoids (mg gFW^{-1})	A (µ mol m^{-2} s^{-1})	E (m mol m^{-2} s^{-1})	gS (m mol m^{-2} s^{-1})
**Control**	0.550 ±0.009	0.087 ±0.006	23.500±0.346	10.977 ±0.515	498.00 ±9.8
**WPL1**	0.541 ±0.029	0.090 ±0.004	22.133 ±2.099	9.907 ±0.915	482.00 ±7.2
**WPL2**	0.568 ±0.018	0.091 ±0.006	24.100 ±2.916	11.400 ±0.500	479.66 ±10.4
**WPL3**	0.556 ±0.017	0.092 ±0.005	21.667 ±2.506	10.053 ±1.132	482.66 ±5.5
**WPL4**	0.589 ±0.014	0.084 ±0.005	21.033 ±0.788	10.573 ±0.179	470.66 ±23.3
**WPL5**	0.588 ±0.048	0.089 ±0.012	24.833 ±0.982	11.583 ±0.642	496.00 ±32.3
**WPL6**	0.545 ±0.008	0.088 ±0.009	22.333 ±1.419	10.903 ±0.205	468.66 ±29.9
**WPL7**	0.575 ±0.037	0.085 ±0.013	21.033 ±1.617	11.440 ±0.662	460.33 ±22.2
**WPL8**	0.569 ±0.080	0.076 ±0.010	21.867 ±1.184	11.990 ±0.746	481.33 ±23.3
**WPS9**	0.551 ±0.021	0.082 ±0.007	23.833 ±1.157	11.417 ±0.761	468.00 ±11.3
**WPS10**	0.586 ±0.014	0.078 ±0.010	22.233 ±0.817	11.550 ±0.658	502.66 ±11.2
**WPS11**	0.591 ±0.045	0.085 ±0.014	21.700 ±0.666	10.253 ±0.636	478.66 ±19.9
**WPR12**	0.567 ±0.038	0.086 ±0.010	24.367 ±0.657	10.780 ±0.850	484.66 ±7.1
**WPR13**	0.517 ±0.015	0.080 ±0.015	22.633 ±1.198	9.510 ±1.124	469.66 ±16.2
**WPR14**	0.491 ±0.058	0.088 ±0.003	24.867 ±0.636	10.647 ±0.840	482.66 ±12.7
**WPR15**	0.576 ±0.033	0.086 ±0.009	23.800 ±2.060	11.983 ±1.059	487.00 ±8.3
**WPR16**	0.615 ±0.028	0.073 ±0.006	23.133 ±1.353	11.447 ±0.578	503.00 ±8.8
**WPR17**	0.539 ±0.075	0.084 ±0.015	24.500 ±0.551	11.543 ±0.501	460.33 ±17.8
**WPR18**	0.581 ±0.033	0.076 ±0.019	24.567 ±0.928	11.353 ±0.553	498.33 ±13.1
**WPR19**	0.620 ±0.057	0.069 ±0.010	23.067 ±3.138	11.490 ±0.734	482.66 ±6.6
**WPR20**	0.612 ±0.058	0.073 ±0.017	25.667 ±1.556	11.210 ±0.527	501.00 ±41.2
**WPR21**	0.593 ±0.040	0.092 ±0.009	21.833 ±1.189	10.313 ±0.458	498.00 ±9.2
**WPS23**	0.588 ±0.069	0.088 ±0.019	22.467 ±0.780	10.430 ±0.482	467.00 ±16.2
**WPR26**	0.597 ±0.027	0.088 ±0.009	22.667 ±1.387	11.347 ±0.690	494.33 ±9.2
**WPR27**	0.627 ±0.058	0.065 ±0.009	21.333 ±1.977	11.643 ±0.328	491.00 ±8.1
**WPR28**	0.553 ±0.035	0.075 ±0.010	23.667 ±1.084	10.390 ±0.366	477.00 ±8.8
**WPR29**	0.622 ±0.041	0.073 ±0.015	24.067 ±1.425	12.113 ±1.170	482.00 ±7.6
**WPR30**	0.631 ±0.019	0.068 ±0.007	21.567 ±1.638	9.967 ±0.882	485.00 ±5.1
**WPR31**	0.568 ±0.059	0.076 ±0.010	21.667 ±1.917	11.947 ±0.802	480.66 ±16.4
**WPR32**	0.573 ±0.027	0.078 ±0.010	23.433 ±1.753	10.477 ±0.424	485.66 ±13.5

A-Net CO_2 assimilation, E-Transpiration rate, gS-Stomatal conductance. Values are the means of six biological replicates ±S.E. Measured parameters were not significantly different at $P \leq 0.05$ according to Duncan's multiple range test.

Supplementary Table S2 Effect of inoculation with isolated bacterial endophytes on shoot and root biomass of *Withania somnifera* plants

Treatment	Shoot weight (g Plant^{-1})	Root weight (g Plant^{-1})
**Control**	18.71 ±2.37	8.55 ±1.13
**WPL1**	17.27 ±0.97	8.07 ±0.75
**WPL2**	18.87 ±2.84	7.66 ±1.04
**WPL3**	17.55 ±1.92	7.92 ±1.18
**WPL4**	18.55 ±2.17	8.28 ±0.64
**WPL5**	17.51 ±0.99	7.18 ±1.04
**WPL6**	18.95 ±2.78	8.20 ±1.05
**WPL7**	18.01 ±1.23	7.75 ±1.45
**WPL8**	16.63 ±1.45	8.32 ±1.14
**WPS9**	18.71 ±2.45	7.66 ±1.23
**WPS10**	18.48 ±0.89	8.94 ±0.54
**WPS11**	17.27 ±1.55	8.07 ±1.04
**WPR12**	17.74 ±2.09	8.71 ±0.97
**WPR13**	18.40 ±0.96	8.78 ±0.83
**WPR14**	16.19 ±1.12	8.37 ±0.61
**WPR15**	17.50 ±1.40	8.75 ±0.88
**WPR16**	17.08 ±1.20	8.57 ±1.28
**WPR17**	17.90 ±1.13	8.76 ±1.35
**WPR18**	19.07 ±2.69	8.79 ±1.27
**WPR19**	17.03 ±1.20	8.26 ±1.38
**WPR20**	18.85 ±1.08	9.48 ±1.41
**WPR21**	18.54 ±1.47	8.68 ±1.62
**WPS23**	16.43 ±1.04	7.06 ±1.57
**WPR26**	16.32 ±2.35	8.07 ±0.98
**WPR27**	18.40 ±1.20	8.15 ±1.06
**WPR28**	16.73 ±1.02	8.71 ±0.77
**WPR29**	18.47 ±0.92	8.41 ±0.52
**WPR30**	17.17 ±1.14	8.15 ±0.79
**WPR31**	17.30 ±1.38	7.42 ±0.57
**WPR32**	16.81 ±1.53	7.91 ±0.92

Values are the means of six biological replicates ±S.E. Measured parameters were not significantly different to the non-inoculated endophyte free control plants at $P \leq 0.05$ according to Duncan's multiple range test.

Supplementary Table S3 Growth promoting characteristics of isolated fungal endophytes

**Strain**	**IAA test**	**Phosphate test**
WPLF1	-	+
WPLF2	-	+
WPLF3	+	+
WPLF4	-	-
WPRF5	-	+
WPRF6	+	-
WPRF7	+	+
WPRF8	-	+
WPRF9	+	+
WPRF10	+	+
WPRF11	+	-

Supplementary Table S4 Growth promoting characteristics of selected bacterial endophytes

Strain	Nitrate test	IAA test	Phosphate test
WPR12	+	+	-
WPR16	+	+	-
WPR17	+	+	-
WPS23	+	+	-
WPR32	+	+	-

Supplementary Table S5 List of primers used for quantitative real time PCR analysis

Gene	Primer	Sequence
FPPS	Forward	5’-CCCTGCTTGTGTTGCTAACG-3’
	Reverse	5’-GCACTGTTTTGCTTGGGTGA-3’
SQS	Forward	5’-ATGGGAACATTGAGGGCGAT-3’
	Reverse	5’-TGCTTCTCCGCATGTCTAGC-3’
SQE	Forward	5’-TCAGGACAATGCCAAACCGA-3’
	Reverse	5’-AAGGGGTGGCGCATATTGAA-3’
CAS	Forward	5’-TTTGTCGGTCCTATCACGCC-3’
	Reverse	5’-TTTGCACACTCATTGCGAGC-3’
SGT	Forward	5’-CGGTTGGGGAAGAGTTTGGA-3’
	Reverse	5’-TGCTCTGATCCAATGCCCTG-3’
HMGR	Forward	5’-CTGGGTTGCCTCTTGATGGT-3’
	Reverse	5’-TGAGCAACAAAGGTCCAGCA-3’
CPR1	Forward	5'- AGCAAGGTATGAGAAGGCAGTTGT -3'
	Reverse	5'- CAGCATTATCAGTTGGCTCACCAT -3'
CPR2	Forward	5'- AGTGTGGCCTAAATTGGATAAGTTGC -3'
	Reverse	5'- ACGATAACATGTCCATTTGCATGAC -3'
DXS	Forward	5’-GCAGAGGGCTTATGACCAGG-3’
	Reverse	5’-ATGCACCGCAATGTGTAGGA-3’
DXR	Forward	5’-CGTCCTGAGTGCAGCAAATG-3’
	Reverse	5’-GACACCAACTCCTCCCGATG-3’
SMT	Forward	5’-CAGCGCTCGAATATGTGGGA-3’
	Reverse	5’-AGTCTGAAATGGGCTTGCGA-3’
ODM	Forward	5’-AAGCGTTTGCCTCCAGTGAT-3’
	Reverse	5’-CACAACCGGTCCCTTGAGAA-3’
Actin	Forward	5'- GAGAGTTTTGATGTCCCTGCCATG -3'
	Reverse	5'- CAACGTCGCATTTCATGATGGAGT -3'

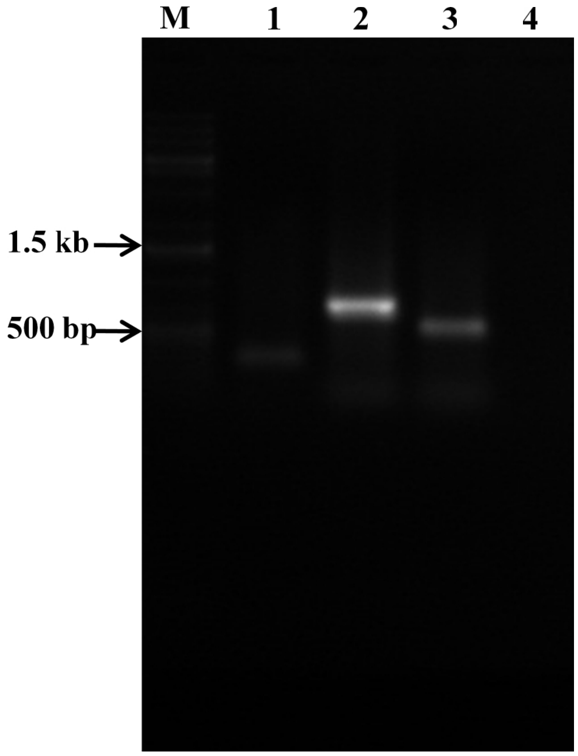

Supplementary Fig. S1: PCR confirmation of *Rol* genes (*Rol A*, *Rol B* and *Rol C*) in transformed root of composite plants. The *Rol* genes integration into the genome of the transformed roots of composite plants was confirmed by genomic DNA-PCR analysis. Expected size of 240 bp (*Rol A*), 762 bp (*Rol B*) and 539 bp (*Rol C*) DNA fragment was observed when PCR amplified using *Rol* genes specific primers while it was absent in the normal plant root. M- Marker, 1- amplification of *Rol A* in composite plant root, 2- amplification of *Rol B* in composite plant root, 3- amplification of *Rol C* in composite plant root, 4- Normal plant root showing absence of *Rol* gene

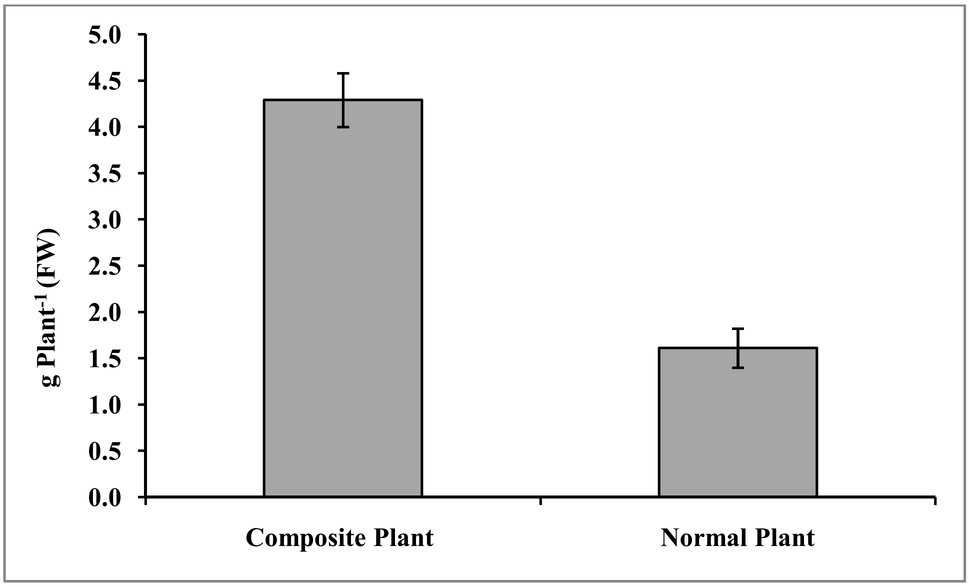

Supplementary Fig. S2: Fresh weight of roots of *in-vitro* grown Normal and Composite *Withania somnifera* plant. Roots of 60 d old *in-vitro* grown Normal and Composite plants were harvested and their fresh weight was measured. Each data point is an average of six replicates and the error bars represent standard errors.

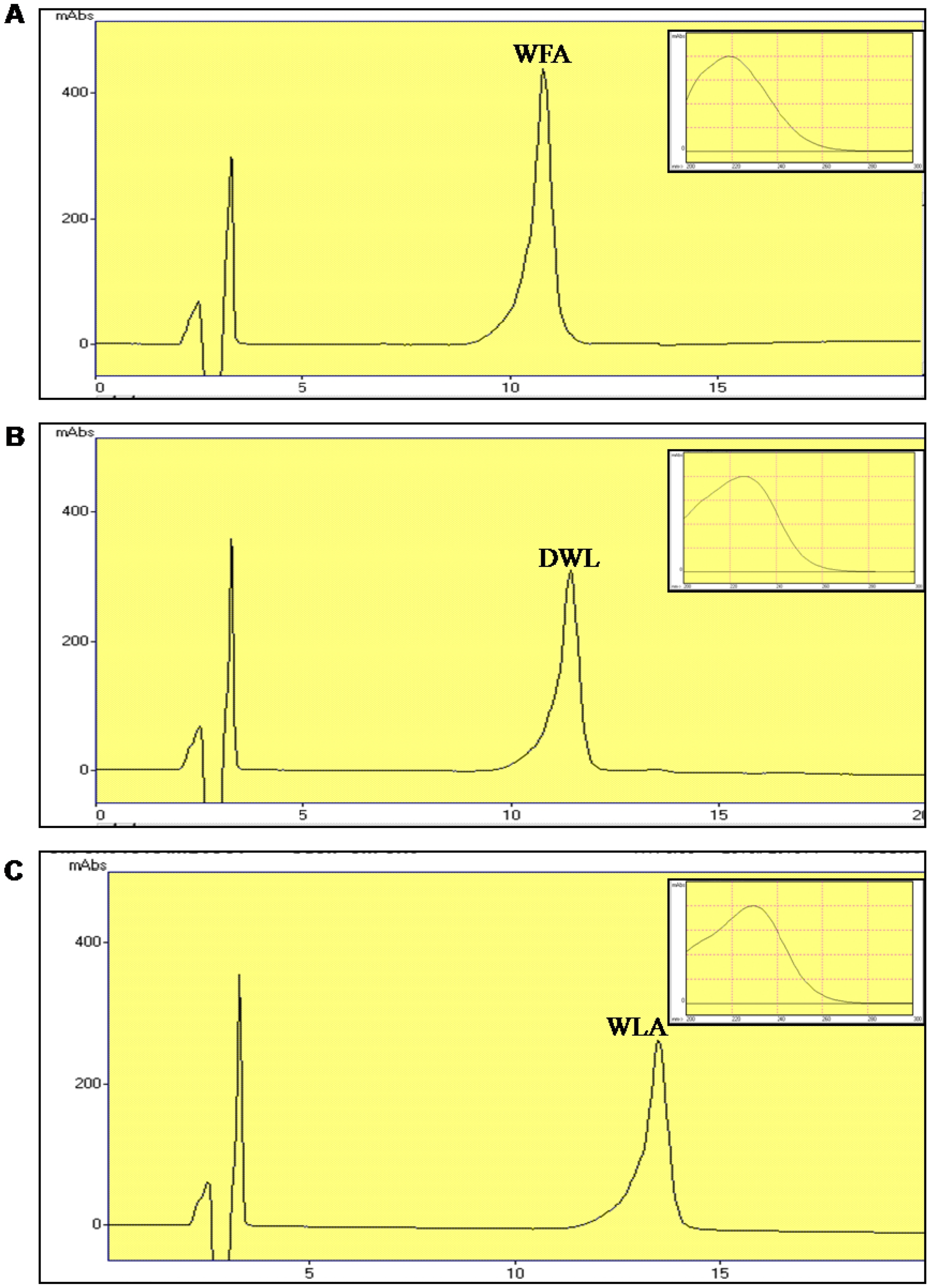

Supplementary Fig. S3: High-performance liquid chromatography elution profile of withanolide standards. (**A**) withaferin A [WFA] (**B**) 12-deoxy withstramonolide [DWL] (**C**) withanolide A [WLA]. Picture in inset represents UV-profile of respective standard.
